# The Association Between Prenatal Maternal Stress and Adolescent Affective Outcomes is Mediated by Childhood Maltreatment and Adolescent Behavioral Inhibition System Sensitivity

**DOI:** 10.1007/s10578-023-01499-9

**Published:** 2023-02-04

**Authors:** T. Sebők-Welker, E. Posta, K. Ágrez, A. Rádosi, E. A. Zubovics, M. J. Réthelyi, I. Ulbert, B. Pászthy, N. Bunford

**Affiliations:** 1 Developmental and Translational Neuroscience Research Group Developmental and Translational Neuroscience Research Group, Research Centre for Natural Sciences, Institute of Cognitive Neuroscience and Psychology, Magyar Tudósok Körútja 2, Budapest, 1117 Hungary; 2 Doctoral School of Mental Health Sciences, Semmelweis University, Balassa U. 6, Budapest, 1083 Hungary; 3 Department of Psychiatry and Psychotherapy, Semmelweis University, Balassa U. 6, Budapest, 1083 Hungary; 4 Integrative Neuroscience Research Group, Research Centre for Natural Sciences, Institute of Cognitive Neuroscience and Psychology, Magyar Tudósok Körútja 2, Budapest, 1117 Hungary; 5 Faculty of Information Technology and Bionics, Pázmány Péter Catholic University, Práter Utca 50/A, Budapest, 1083 Hungary; 6 1st Department of Paediatrics, Semmelweis University, Bókay János U. 53-54, Budapest, 1083 Hungary

**Keywords:** Prenatal maternal stress, Adolescent, Transdiagnostic, Affectivity, Aggression, Emotion regulation, Childhood maltreatment, Behavioral inhibition system

## Abstract

Prenatal maternal stress is linked to offspring outcomes; however, there is little research on adolescents, behavioral, transdiagnostic outcomes, or the mechanisms through which relations operate. We examined, in *N* = 268 adolescents (*M*_age_ = 15.31 years; SD = 1.063; 57.8% boys) whether prenatal maternal stress is associated with adolescent affective outcomes; whether this association is mediated, serially, by childhood home atmosphere and adolescent behavioral inhibition system (BIS) sensitivity; and whether mediational effects are moderated by adolescent attention-deficit/hyperactivity disorder or maternal internalizing symptomology. Prenatal maternal daily stress and major life events were associated with adolescent outcomes through childhood negative atmosphere/neglect and BIS sensitivity, with no evidence of moderation. Results have implications regarding the effect of prenatal maternal stress on offspring outcomes and regarding corresponding sensitive periods.

**Supplementary Information:**

The online version contains supplementary material available at 10.1007/s10578-023-01499-9.

## Introduction

Across the globe, the prevalence of pediatric mental health disorders and problems has been increasing [1] and currently, up to 20% of adolescents experience a mental health disorder, with the third leading cause of death among adolescents (ages 15–19 years) being suicide [2]. The personal and societal burden of mental disorders are great; 7% of all burden of disease as measured in disability adjusted life years (DALYs) and 19% of all years-lived-with-disability are due to such disorders [3]. Of note, 50% of mental health disorders develop by the age of 14 years and for 75% of mental disorders, age-of-onset is before 24 years [4]. Accordingly, international policy guidelines underscore the importance of early identification and prevention of precursors or signs of psychopathology. Early intervention is cost-effective, can improve adult economic productivity [5] and adult health [6], as well as lessen the risk of adult psychopathology [7].

A sensitive and relevant developmental period in this regard is the prenatal period; as a result of the brain’s plasticity and sensitivity to environmental influences, prenatal maternal experiences can affect offspring development and functioning [8]. For example, prenatal maternal pharmacological treatment, maternal exposure to various chemical and non-chemical stressors (e.g., NO_2_, opioids), maternal preconception and prenatal nutrition are each associated with childhood cognitive outcomes [9], externalizing behaviors [10], developmental disorders [11] and neurodevelopment [12], and motor development [13]. Another aspect of the prenatal period, maternal exposure to stress, has also been linked to offspring outcomes. In animals, a large body of research indicates prenatal maternal stress affects offspring brain development, across preclinical [14–16] and non-human primate studies [17, 18]. Specifically, in utero stress exposure affects amygdala, corpus callosum, frontal cortex, hippocampus, and rostral anterior cingulate cortex development [19] and, in accordance with the functional neuroanatomy of these regions, results in attention deficits and behaviors linked to anxiety and depression (e.g., learned helplessness and alterations in circadian rhythm) [19]. Notably, in animals, prenatal stress effects endure throughout the lifespan [20]. In humans, although there are considerable differences across studies in methodology, e.g., with regard to the operationalization of stress^1^ and also in terms of the exact findings, the literature is consistent in indicating that prenatal maternal stress is associated with increased risk for offspring behavioral, cognitive, and mental health problems [21]. Difficulties include cognitive deficits, a difficult temperament (low effortful control, high negative affectivity, and atypical reward sensitivity) and psychiatric disorders [21–23].

Regarding pathways, although the precise mechanisms via which gestational stress is associated with neurodevelopment and increases risk for offspring behavioral and cognitive problems have yet to be elucidated [19], prenatal maternal stress (and its proxies, such as anxiety and depression) impact development of the amygdala, limbic system, frontal lobes [24–27] and, via fetal exposure to elevated cortisol, brain systems related to stress processing and regulation, i.e., the hypothalamus [22] and hypothalamic-pituitary (HPA) axis [28] as well as the septohippocampal system [29].

The HPA axis is a neuroendocrine axis comprised of a brain system (the hypothalamus), the non-neural part of the pituitary (adenohypophysis) and the adrenal cortex. These interact with each other via a complex set of direct influences and feedback interactions to regulate bodily reactions to stress. The hypothalamus releases corticotrophin-releasing factor (CRF), thereby affecting the pituitary gland, which, in response releases adrenocorticotropic hormone (ACTH), which causes the adrenal cortex to release glucocorticoids, e.g., cortisol. Cortisol, in turn, affects HPA axis regulation [30]. Prenatal maternal stress, via hypoactivity of a placental enzyme 11-beta hydroxysteroid dehydrogenase-2 (11ß-HSD2) responsible for regulating the amount of cortisol passing through the placenta, may result in elevated fetal exposure to cortisol [31–33] and this may affect fetal HPA-axis development and results in offspring HPA-axis hyperresponsiveness [34]. The septohippocampal system comprises the hippocampus and the septum [35] and is implicated in the resolution of goal conflict [36]. Specifically, the septohippocampal system, and its monoaminergic brainstem afferents are implicated in sensitivity of the behavioral inhibition system (BIS) [36, 37].

The BIS is part of a larger architecture of attention- and motivation-regulating systems involving the behavioral activation system (BAS), the BIS, and the fight/flight/freeze system (FFFS) [38]. The functioning of this architecture of systems rests on functional distinctions between behaviors; e.g., behaviors that remove an organism from a source of danger (e.g., flight, fight, or freezing), a function governed by the FFFS, are different from those that allow it to assess a potential source of danger so as to determine an appropriate response, a function governed by the BIS [29]. As such, the BIS is a conflict detecting, monitoring, and resolving system that functions as a *comparator* of inputs to determine course of action [29, 36, 37]. Accordingly, although termed the ‘behavioral inhibition system’, the BIS both inhibits pre-potent behavior and generates additional outputs of attention and arousal to support exploratory behavior designed to resolve conflict [29]. Therefore, a function of the BIS is making assessments in situations involving approach-avoidance, approach-approach, and avoidance-avoidance conflicts [29, 36, 37]. The BIS is not only associated with anxiety and stress but its sensitivity is positively associated with ‘neuroticism’, or variously termed negative affectivity (NA) [39], i.e., the stable tendency to experience negative emotions [40].

Taken together, evidence indicates maternal prenatal stress is associated with offspring outcomes, and it stands to reason that the former exerts its effects on the latter including via its impact on the offspring stress systems, such as the septohippocampal system and its bio-behavioral correlate, the BIS. However, gaps in knowledge remain.

*First*, focus with regard to offspring outcomes has mostly been on infants and young children, without consideration of long-term effects observable in adolescence. Adolescence is a sensitive period from the perspective of brain development, as during this developmental period, enhanced neuroplasticity and structural and functional changes confer both advantages (e.g., skill learning facility) [41] and vulnerabilities (e.g., psychopathology risk) [41, 42]. Adolescent events of neuromaturation indicate this developmental phase may be a relatively sensitive window into the effects of earlier influences on neurodevelopment; adolescent developmental processes may reveal differences that were masked previously [43]. Better understanding the relation between prenatal maternal stress and adolescent offspring outcomes may be particularly relevant in this regard, as a host of mental health difficulties emerge in adolescence [44]. Early life adversity may set forth a “cascade” of molecular, cellular and/ or network-level effects in the developing brain [19, 45, 46], yet these may not manifest until later [47–49], such as in adolescence. In studies where adolescent or adult outcomes of prenatal maternal stress were examined, associations and differences on both a neural and a behavioral level were observed. On a neural level, prenatal maternal stress has been linked to adolescent offspring lower overall grey matter volume, especially in cortical regions associated with depression [50], adolescent offspring enhanced event-related potentials during endogenous cognitive control [51], and young adult offspring enhanced functional brain connectivity (which, in turn, correlated with depressive symptoms) [52]. On a behavioral level, prenatal maternal stress has been related to both higher externalizing [53, 54] and internalizing [53, 55–57] symptoms, ADHD and impulsivity [58–60] and differences in endogenous/exogenous control [51, 61].

*Second*, although there has been focus on offspring externalizing and internalizing disorders, there is a relative paucity of studies focused on transdiagnostic characteristics (but see the studies by [50] indicating prenatal maternal stress was associated with adult offspring affect dysregulation; by [51, 61, 62] and [63] indicating prenatal maternal stress was associated with differences in neuropsychological outcomes including inhibition and working memory). Transdiagnostic characteristics would be key to study in relation to prenatal maternal stress, as they are applicable, beyond clinical samples, to the general population and might result in adverse outcomes for youth, even in the absence of a diagnosable psychopathology [22].

*Third*, there are examinations of biological moderators (e.g., 5-HTTLPR [64] and mediators (e.g., DNA methylation [65–67], glucocorticoids [68]) of the link between prenatal maternal stress and offspring biological outcomes (i.e., offspring BMI and central adiposity [66], c-peptide secretion [67], cytokine production [65]). However, when it comes to individual differences in *bio-behavioral and affective functioning*, the majority of the literature is focused on bivariate associations, without consideration of boundary conditions (moderators; for whom or when effects operate) and mechanisms (mediators; how effects operate) of the relation between prenatal maternal stress and offspring outcomes [69]. Exceptions are a handful of studies assessing moderation, with mixed results. Across these studies, moderators tested were brain-derived neurotrophic factor (BDNF) genotype [57], COMT genotype [60], serotonin transporter polymorphism 5-HTTLPR [64] and maternal stress [70]. Outcomes of interest were offspring ADHD symptoms/working memory [60], behavioral disturbance [64], and internalizing symptoms [57, 70]. Across studies, the predictor was prenatal maternal anxiety [70]. Further exceptions are a comparably small number of investigations of mediation. Mediators tested were offspring childhood maltreatment [71, 72], executive functioning [73], HPA axis regulation [74], and temperamental negativity [75], as well as maternal general anxiety and mindful parenting [69]. Outcomes were offspring emotion regulation at three and 6 months of age [75]; internalizing problems in childhood [69]; academic achievement [73], antisocial behavior [72], and depression [74] in adolescence; and depression in adulthood [71]. Across studies, the predictor was prenatal maternal anxiety and/or depression [72].

To begin filling gaps in knowledge about adolescent affective outcomes of prenatal maternal stress, including more minor stress (and not only at more severe levels, as in anxiety and depression), with consideration of bio-behavioral mechanisms, our aim in the current study was to examine the relations between prenatal maternal stress, indices of adolescent emotion processing, specifically, affectivity, aggression, and emotion regulation, and how individual differences in adolescent BIS sensitivity affect these relations.

Beyond BIS sensitivity however, a conceptually and empirically relevant variable that prenatal maternal stress is related to and that might mediate or modulate the effects of such stress is the postnatal/early childhood home environment. Data show, for example, a positive association between prenatal stress and postnatal hostile-reactive parenting (Hentges et al. 2019) and that compared to non-exposed offspring, adult offspring exposed to prenatal maternal depression are over twice as likely to have experienced child maltreatment (Plant et al. [71]).

The negative effects of prenatal maternal stress through the postnatal home environment may operate through a diathesis-stress effect where negative outcomes result from an interaction between a predispositional vulnerability, i.e., the diathesis (e.g., prenatal maternal stress), and stress caused by life experiences (e.g., negative postnatal home environment) [76–78].

An alternative is that prenatal maternal stress may not only function as a diathesis but also as a source of developmental plasticity [78]. The differential susceptibility hypothesis extends the diathesis-stress model in positing that not only are certain individuals more vulnerable to the “risk” effects of negative environments but that certain individuals are (also) more vulnerable to the “protective effects” of positive environments, i.e., they are developmentally plastic [79]. Experimental findings with animals and observational data with humans support this hypothesis [71, 78, 80–83]. In humans, data show negative affectivity and physiological reactivity are well documented consequences of prenatal maternal stress. Individuals high on negative affectivity and physiological reactivity, when in aversive rearing environments, exert greatest deficits and difficulties across a variety of behavioral and psychological phenotypes but when in a supporting environment, exert greatest benefits on such measures (with individuals lower on negative affectivity falling in-between these extremes) [78].

In addition to findings indicating an association between prenatal maternal stress and postnatal home environment, results also suggest childhood maltreatment may affect child reinforcement sensitivity and emotion regulation; results show that young adults who were chronically mistreated as children exhibit greater threat sensitivity than their nonchronically mistreated or non-mistreated counterparts (Thompson et al. 84). Also, adults with a history of childhood interpersonal trauma exhibit higher punishment and lower reward sensitivity as well as greater BIS sensitivity (Miu et al. [85]). Accordingly, it stands to reason that prenatal maternal stress exerts its influence on adolescent outcomes through the early childhood environment which, in turn, alters the child’s reinforcement, i.e., BIS sensitivity, which, in turn is what results in negative outcomes.

## Current Study

Our aims in the current study were to examine, for the first time, in a large sample of middle-late adolescents, whether (1) prenatal maternal stress—using measures of both more minor, daily stressors and of more major, life events stressors—is associated with indices of emotion processing, specifically, affectivity, aggression, and emotion regulation and (2) these associations are mediated, serially, by differences in the postnatal home environment and by reinforcement sensitivity, indexed by behavioral inhibition system (BIS) sensitivity. (3) Furthermore, given that parental psychopathology may affect the examined relations, insofar as it may affect the postnatal home environment, e.g., in the form of parenting style [86], and as it is a proxy for genetic predisposition to offspring psychopathology [87], our goal was to examine whether any of the mediational effects are moderated by maternal internalizing problems.

## Method

### Procedures

Data were collected in the context of a larger longitudinal project, the (BLINDED) study, aimed at identifying behavioral and biological protective and risk factors of behavior problems and functional impairments in adolescents exhibiting a range of attention-deficit/hyperactivity disorder (ADHD) symptoms but oversampled for ADHD. Data were obtained during the second year (baseline assessments) in the current study.

Adolescents between the ages of 14 and 17 years were recruited mainly from public middle-, technical and vocational-, and high schools as well as two child and adolescent psychiatry clinics in Budapest, Hungary. In case of schools, research staff visited classrooms and presented on the opportunity to participate in a research program. In case of clinics, research staff distributed an e-mail and fliers with information on the research program. Exclusionary criteria were cognitive ability at or below the percentile rank corresponding to an FSIQ of 80 across administered indices; autism spectrum disorder (severity ≥ 2); neurological illness; and having visual impairment as defined by impaired vision < 50 cm, unless corrected by glasses or contact lenses.

Parents and participants (i.e., adolescents) provided written informed consent (and assent). Adolescents underwent a series of tests, including assessment of cognitive ability and a structured clinical interview, followed by buccal swab and passive drool genetic sampling, and completion of questionnaires across two sessions. Self-report questionnaires completed by adolescents (detailed below) were assessments of affectivity, emotion dysregulation, reinforcement sensitivity, aggression and of the early childhood home environment. Questionnaires completed by parents involved parent-report measures of adolescent behavior and functioning (including ADHD) and parental self-report measures of prenatal maternal stress and parental psychopathology. All questionnaires were completed digitally, on a computer or tablet, using the Psytoolkit platform [88, 89] and the Qualtrics software, Version June 2020–March 2021 (Qualtrics, Provo, UT). This research was approved by the National Institute of Pharmacy and Nutrition (OGYÉI/17089-8/2019) and has been performed in accordance with the ethical standards laid down in the 1964 Declaration of Helsinki and its later amendments.

All (but ADHD and externalizing) diagnoses were determined using a combination of the Mini-International Neuropsychiatric Interview for Children and Adolescents (MINI Kid) [90] and the Structured Clinical Interview for DSM-5 Disorders, Clinical Version (SCID-5 CV) [91]. ADHD diagnoses were determined using parent-report on the ADHD Rating Scale-5 (ARS-5) [92]. For an ADHD diagnosis, adolescents had to meet a total of five (in case of youth < 17 years old) or six (in case of youth ≥ 17 years old) (or more) of the Diagnostic and Statistical Manual of Mental Disorders (5th ed; DSM-5) [93] inattention or hyperactivity/impulsivity symptoms and exhibit impairment (i.e., rating of 2 = moderate impairment or 3 = severe impairment) in at least three areas of functioning (G. DuPaul, personal communication, July 19, 2021). Unanimous agreement by two licensed child psychiatrists was required for ADHD diagnoses.

### Participants

Participants included in the current study were 268 adolescents between the ages of 14 and 18 years (*M*_age_ = 15.31 years; SD = 1.063; 57.8% boys), *n* = 66 met criteria for ADHD. The majority (96.3%) identified as White and 3.7% identified as part of an ethnic minority group in Hungary. Average cognitive ability was in the 61.79th percentile (SD = 20.86), with estimated VCI percentile rank: *M* = 66.42, SD = 22.98, estimated PRI percentile rank *M* = 57.16, SD = 26.33. Participants were from an above-average socioeconomic background based on parental income (average family net income fell in the 5 001–700 000 HUF/month range, with average net income in Hungary being 289 000 HUF/month) [94].

### Measures

For detailed description of measures, see Supplement. Cognitive functioning was estimated using abbreviated versions of the Wechsler Intelligence Scales [95, 96]. Minor prenatal stress was measured using the perceived stress scale (PSS) [97] and major prenatal stress using the prenatal life event scale (PLES) [98]. Affectivity, aggression, and emotion regulation were assessed via the positive and negative affect schedule (PANAS) [99], the difficulties in emotion regulation scale (DERS) [100] and the Buss-Perry aggression questionnaire (BPAQ) [101], respectively. The postnatal home environment was measured with the Neglect/Negative Home Atmosphere subscale of the child abuse and trauma scale (CATS) [102]. BIS sensitivity was measured with the BIS subscale of the Reinforcement Sensitivity Theory of Personality Questionnaire (RST-PQ) [103]. Maternal internalizing problems were assessed on the Internalizing Problems subscale of the Adult Self-Report 18–59 [104]. Adolescent ADHD status was determined using the home version of the ADHD rating scale-5 (ARS 5) [92].

### Analytic Plan

We collected questionnaire data digitally, which requested a response for all questions. Due to sensitive issues, however, items from the CATS questionnaire were not mandatory, thus data on some items were missing for some participants; of the 38 CATS items 24 had missing data from ≥ one participant. Of the 268 adolescents, 242 responded to all CATS questions and 9 had missing data on ≥ one (typically, one to four) item, with a total of 88 missing CATS data points. Multiple imputation was used to substitute missing data; consistent with this method, five alternative iterations were created for possible responses to missing data points and the mean of these five iterations were used for imputation.

To examine the association among study variables, bivariate correlations were computed. To examine whether associations between prenatal maternal stress (as indexed by maternal report on the PLES and PSS) and adolescent offspring outcomes of interest (i.e., self-report affectivity, ED, and aggression) are mediated by childhood maltreatment variables (as indexed by CATS subscales) and BIS sensitivity as serial mediators, we used PROCESS Version 3.5 [105] to calculate 95% CIs around the indirect effect with 5000 bootstrap resamples,^2^ implementing a heteroscedasticity-consistent standard error estimator. For all mediation findings, we report the completely standardized indirect effect(s).

Bivariate correlations were repeated with the subsample where the mother completed the Adult Self-Report (as interest was in maternal internalizing symptoms; *n* = 244). In addition to maternal internalizing psychopathology, as (1) our sample was oversampled for ADHD and (2) there are sex differences in the effects of prenatal stress [106], and sex differences in the incidence and prevalence of mental disorders begin to emerge during adolescence [107, 108], we also examined whether adolescent ADHD status and sex moderate the mediational effect of CATS neglect/negative atmosphere and BIS sensitivity. To this end, we conducted follow-up moderated mediational analyses in case of models that were supported in the serial mediational analyses. Adult Self-Report Internalizing Problems T Score, adolescent ADHD diagnosis, and adolescent sex were examined as moderators of the indirect path (from prenatal maternal stress to adolescent outcomes through CATS neglect/negative atmosphere and BIS sensitivity) and the direct path (from prenatal maternal stress to adolescent outcomes) in the mediational model, also using PROCESS Version 3.5 [105] and 5000 bootstrap resamples, implementing a heteroscedasticity-consistent standard error estimator.

For all mediation findings, we report the completely standardized indirect effect(s). Indirect effect 1 corresponds to the effect of the prenatal maternal stress variable(s) on the outcome through childhood maltreatment variable(s), Indirect effect 2 corresponds to the effect of the prenatal maternal stress variable(s) on the outcome through BIS sensitivity, and Indirect effect 3 corresponds to the serial mediation effect, i.e., the effect of the prenatal maternal stress variable(s) on the outcome through childhood maltreatment variable(s) and BIS sensitivity operating serially.

## Results

### Descriptive Analyses

Regarding prenatal maternal substance use, 19 (7.09%) mothers reported any kind of substance use while pregnant; 70 (26.12%) mothers reported their child was not born to term (i.e., was more than a week either pre-term or late term), 81 (30.22%) reported they experienced some form of complication during or after giving birth, 70 (26.12%) reported they had a high risk pregnancy, 18 (6.72%) reported they had an inflammatory disease while pregnant, and 16 (5.97%) reported their child was born with a lower than average birth weight (see Supplementary Table S1).

On the PLES, items most frequently endorsed and rated as at least “moderately negative or undesirable” were: “*13. Did you have serious arguments several times with someone?*” (*n* = 44 [16.30%]); “*9. Did you have unusual financial pressures or trouble with money?*” (*n* = 32 (11.80%)); “*24. During your pregnancy, did you or a close family member or friend experience serious physical injury, illness, or hospitalization?*” (*n* = 27 (10%)); “*8. Did you have unusually big pressures or conflicts at work?*” (*n* = 22 (8.20%)); (see Supplementary Table S2).

On the PSS, items most frequently endorsed as at least ‘fairly often’ (or ‘sometimes’ in case of reverse-scored items) were: “*12. found yourself thinking about things that you have to accomplish?*” (*n* = 105 (38.90%)); “*1. been upset because of something that happened unexpectedly?*” (*n* = 47 (17.40%)); “*3. felt nervous and “stressed”?*” (*n* = 41 (15.20%)); “*8. found that you could not cope with all the things that you had to do?*” (*n* = 40 (14.90%)); “*11. been angered because of things that were outside of your control?” *(*n* = 40 (14.80%)); (see Supplementary Table S2).

### Bivariate Correlation Analyses

The prenatal maternal stress variables were positively correlated, with the correlation coefficient corresponding to a medium effect (Table 1). Greater PLES scores were associated with greater CATS neglect/negative atmosphere and BIS sensitivity—all small effects; PSS scores were associated with these variables as well as positively associated with greater ED and NA—also a small effect (Table 1). The two hypothesized mediators, CATS neglect/negative atmosphere and BIS sensitivity were positively correlated with each other, with the correlation coefficient corresponding to a medium effect, and both were positively correlated with all hypothesized outcomes (i.e., NA, ED, and aggression—CATS neglect/negative atmosphere and outcomes: medium to large effects and BIS sensitivity and outcomes: also medium (aggression) to large (NA, ED) effects) indicating the proposed mediator-outcome combinations are suitable for mediation analysis [109] (Table 1). NA, ED, and aggression were also positively related—all medium to large effects (Table 1). Girls reported greater CATS neglect/negative atmosphere, ED, NA and greater BIS sensitivity and older age was associated with greater ED, NA and BIS sensitivity (small effects).

**Table 1:** Bivariate correlations among study variables

			1	2	3	4	5	6	7	8	9
1. ADHD status	*r (p)*		–								
	Bootstrap	Bias (SE)	–								
		95% CI	–								
2. ED	*r (p)*		**0.196** (0.002)	–							
	Bootstrap^c^	Bias (SE)	0.000 (0.064)	–							
		95% CI	0.074; 0.322	–							
3. NA	*r (p)*		**0.162** (0.009)	**0.709** (< 0.001)	–						
	Bootstrap^c^	Bias (SE)	0.001 (0.066)	0.001 (0.037)	–						
		95% CI	0.029; 0.290	0.625; 0.774	–						
4. CATS NNA	*r (p)*		**0.128** (0.040)	**0.494** (< 0.001)	**0.502** (< 0.001)	–					
	Bootstrap^c^	Bias (SE)	0.000 (0.060)	− 0.001 (0.064)	0.000 (0.059)	–					
		95% CI	0.000; 0.252	0.366; 0.614	0.381; 0.614	–					
5. Aggression	*r (p)*		0.055 (0.380)	**0.392** (< 0.001)	**0.457** (< 0.001)	**0.346** (< 0.001)	–				
	Bootstrap^c^	Bias (SE)	0.001 (0.067)	0.003 (0.058)	0.002 (0.055)	0.004 (0.064)	–				
		95% CI	− 0.077; 0.193	0.277; 0.502	0.347; 0.564	0.224; 0.469	–				
6. BIS	*r (p)*		0.024 (0.705)	**0.707** (< 0.001)	**0.702** (< 0.001)	**0.512** (< 0.001)	**0.355** (< 0.001)	–			
	Bootstrap^c^	Bias (SE)	0.001 (0.067)	0.001 (0.033)	0.001 (0.031)	0.000 (0.056)	0.002 (0.065)	–			
		95% CI	− 0.100; 0.158	0.636; 0.769	0.644; 0.761	0.398;0.614	0.219; 0.482	–			
7. PSS	*r (p)*		**0.307** (< 0.001)	**0.255** (< 0.001)	**0.170** (0.006)	**0.233** (< 0.001)	0.009 (0.892)	**0.174** (0.005)	–		
	Bootstrap^c^	Bias (SE)	− 0.002 (0.059)	0.000 (0.057)	0.003 (0.060)	0.002 (0.058)	0.000 (0.063)	0.000 (0.057)	–		
		95% CI	0.190; 0.425	0.137; 0.365	0.052; 0.291	0.122; 0.345	− 0.115; 0.131	0.060; 0.280	–		
8. PLES	*r (p)*		**0.133** (0.032)	0.113 (0.070)	0.116 (0.063)	**0.130** (0.038)	0.002 (0.973)	**0.124** (0.047)	**0.384** (< 0.001)	–	
	Bootstrap^c^	Bias (SE)	0.001 (0.066)	0.003 (0.058)	0.003 (0.052)	0.004 (0.056)	0.000 (0.062)	0.004 (0.063)	0.000 (0.058)	–	
		95% CI	0.002; 0.265	0.004; 0.223	0.015; 0.225	0.029; 0.248	− 0.117; 0.130	0.000; 0.244	0.276; 0.497	–	
9. Age	*r (p)*		− 0.019 (0.758)	**0.130** (0.038)	**0.144** (0.021)	0.073 (0.246)	0.096 (0.124)	**0.223** (< 0.001)	−0.045 (0.478)	− 0.064 (0.308)	–
	Bootstrap^c^	Bias (SE)	0.000 (0.063)	− 0.001 (0.058)	0.000 (0.060)	− 0.001 (0.062)	0.000 (0.060)	− 0.001 (0.059)	0.000 (0.059)	0.001 (0.063)	–
		95% CI	− 0.140; 0.108	0.019; 0.242	0.020; 0.263	− 0.046; 0.202	− 0.020; 0.213	0.103; 0.336	− 0.156; 0.072	− 0.183; 0.062	–
10. Sex	*r (p)*		**− 0.136** (0.029)	**0.180** (0.004)	**0.161** (0.010)	**0.272** (< 0.001)	− 0.060 (0.337)	**0.273** (< 0.001)	0.083 (0.186)	**− 0.242** (< 0.001)	− 0.067 (0.283)
	Bootstrap^c^	Bias (SE)	0.000 (0.061)	− 0.001 (0.059)	− 0.003 (0.060)	− 0.001 (0.055)	− 0.004 (0.063)	− 0.003 (0.057)	0.000 (0.064)	− 0.001 (0.057)	0.000 (0.062)
		95% CI	− 254; − 0.011	0.060; 0.293	0.038; 0.274	0.157; 0.376	− 0.194; 0.063	0.155; 0.386	− 0.048; 0.206	− 0.348; − 0.121	− 0.183; 0.056

Significant correlations are in bold

*PLES* prenatal life events scale, *PSS* perceived stress scale, *CATS NNA* child abuse and trauma survey, neglect/negative home atmosphere subscale, *BIS* behavioral inhibition system, *NA* negative affectivity, *ED* emotion dysregulation

When repeating bivariate correlations restricted to the portion of the sample whose mothers completed the Adult Self-Report (as interest was in maternal internalizing symptoms; *n* = 244), the pattern of correlations among hypothesized predictor, mediator, outcome, sex, and age variables was the same as in the overall sample (Table 2). Maternal internalizing symptoms were positively associated with PLES (*r* = 0.229, *p* < 0.001) and PSS (*r* = 0.475, *p* < 0.001) scores (a small and a medium effect, respectively) and also with adolescent offspring BIS sensitivity (*r* = 0.236, *p* < 0.001) and NA (*r* = 0.256, *p* < 0.001) (medium effects) and ED (*r* = 0.253 *p* < 0.001) (a borderline medium effect) and CATS neglect/negative atmosphere (*r* = 0.168 *p* = 0.009) (Table 2).

**Table 2:** Bivariate correlations among study variables restricted to a subsample (*n* = 244) with available maternal internalizing symptoms data

				1	2	3	4	5	6	7	8	9	10
1. ADHD status		*r (p)*		–									
		Boot	Bias (SE)	–									
			95% CI	–									
2. Int		*r (p)*		**0.229** (< 0.001)	–								
		Boot	Bias (SE)	− 0.001 (0.062)	–								
			95% CI	0.108; 0.344	–								
3. Age		*r (p)*		− 0.001 (0.993)	− 0.049 (0.447)	–							
		Boot	Bias (SE)	− 0.004 (0.063)	0.002 (0.068)	–							
			95% CI	− 0.129; 0.113	− 0.179; 0.088	–							
4. Sex		*r (p)*		**− 0.126** (0.049)	0.049 (0.447)	− 0.086 (0.183)	–						
		Boot	Bias (SE)	0.001 (0.062)	0.002 (0.061)	0.000 (0.064)	–						
			95% CI	− 0.241; − 0.004	− 0.064; 0.175	− 0.216; 0.042	–						
5. ED		*r (p)*		**0.228** (< 0.001)	**0.253** (< 0.001)	**0.140** (0.028)	**0.193** (0.002)	–					
		Boot	Bias (SE)	0.003 (0.069)	0.002 (0.061)	0.001 (0.063)	− 0.001 (0.061)	–					
			95% CI	0.095; 0.368	0.135; 0.372	0.015; 0.271	0.063; 0.310	–					
6. NA		*r (p)*		**0.190** (0.003)	**0.256** (< 0.001)	**0.148** (0.021)	**0.166** (0.009)	**0.708** (< 0.001)	–				
		Boot	Bias (SE)	0.000 (0.068)	− 0.004 (0.064)	− 0.001 (0.063)	− 0.001 (0.064)	− 0.002 (0.038)	–				
			95% CI	0.056; 0.324	0.117; 0.371	0.022; 0.271	0.026; 0.281	0.630; 0.774	–				
7. CATS NNA		*r (p)*		**0.125** (0.051)	**0.168** (0.009)	0.085 (0.186)	**0.276** (< 0.001)	**0.506** (< 0.001)	**0.513** (< 0.001)	–			
		Boot	Bias (SE)	0.001 (0.071)	−0.002 (0.055)	0.000 (0.066)	0.001 (0.057)	− 0.002 (0.065)	− 0.003 (0.060)	–			
			95% CI	− 0.017; 0.268	0.060; 0.266	− 0.050; 0.209	0.161; 0.385	0.366; 0.621	0.383; 0622	–			
8. Agg		*r (p)*		0.084 (0.190)	−0.019 (0.767)	0.109 (0.090)	− .073 (0.253)	**0.383** (< 0.001)	**0.450** (< 0.001)	**0.358** (< 0.001)	–		
		Boot	Bias (SE)	0.003 (0.069)	0.000 (0.070)	−0.001 (0.064)	− 0.001 (0.063)	−0.002 (0.063)	− 0.002 (0.057)	0.000 (0.065)	–		
			95% CI	− 0.044; 0.225	− 0.149; 0.124	− 0.027; 0.230	− 0.197; 0.050	0.256; 0.495	0.325; 0.555	0.234; 0.487	–		
9. BIS		*r (p)*		0.049 (0.445)	**0.236** (< 0.001)	**0.223** (< 0.001)	**0.272** (< 0.001)	**0.712** (< 0.001)	**0.705** (< 0.001)	**0.531** (< 0.001)	**0.340** (< 0.001)	–	
		Boot	Bias (SE)	0.001 (0.073)	−0.001 (0.060)	0.000 (0.062)	−0.002 (0.059)	−0.002 (0.034)	− 0.001 (0.031)	− 0.001 (0.056)	0.000 (0.069)	–	
			95% CI	− 0.099; 0.192	0.115; 0.347	0.102; 0.348	0.146; 0.380	0.636; 0.768	0.639; .760	0.413; 0.630	0.200; 0.472	–	
10. PSS		*r (p)*		**0.298** (< 0.001)	**0.475** (< 0.001)	− 0.039 (0.543)	0.109 (0.088)	**0.288** (< 0.001)	**0.189** (0.003)	**0.244** (< 0.001)	0.036 (0.580)	**0.198** (0.002)	–
		Boot	Bias (SE)	0.002 (0.060)	− 0.002 (0.050)	− 0.001 (0.063)	− 0.001 (0.062)	− 0.001 (0.055)	− 0.002 (0.060)	− 0.004 (0.063)	− 0.002 (0.065)	− .002 (0.057)	–
			95% CI	0.181; 0.410	0.374; 0.567	− 0.156; 0.094	− 0.017; 0.234	0.177; 0.398	0.063; 0.303	0.119; 0.364	− 0.096; 0.157	0.084; 0.307	–
11. PLES	*r (p)*			0.114 (0.074)	**0.229** (< 0.001)	− 0.057 (0.378)	0.009 (0.890)	0.124 (0.053)	**0.131** (0.040)	0.125 (0.051)	0.037 (0.565)	**0.178** (0.005)	**0.387** (< 0.001)
	Boot		Bias (SE)	0.000 (0.071)	0.002 (0.055)	0.001 (0.067)	− 0.002 (0.064)	0.001 (0.057)	0.000 (0.055)	0.000 (0.061)	0.000 (0.066)	− 0.001 (0.060)	0.003 (0.058)
			95% CI	− 0.025; 0.260	0.122; 0.338	− 0.181; 0.080	− 0.115; 0.139	0.019; 0.242	0.029; 0.246	0.014; 0.256	− 0.091; 0.170	0.057; 0.297	0.272; 0.501

Significant correlations are in bold

*Boot* bootstrap, *Int* maternal anxiety and depression problems as indexed by the Adult Self-Report Form, Internalizing Problems T score, *ED* emotion dysregulation as measured by the difficulties in emotion regulation scale, *NA* negative affectivity, *CATS NNA* child abuse and trauma scale–neglect/negative home atmosphere subscale, *Agg* aggression, *BIS* behavioral inhibition system, *PSS* perceived stress scale, *PLES* prenatal life events scale

### Mediation Analyses with the PLES as the Predictor

#### Negative Affectivity as the Outcome

CATS neglect/negative atmosphere (NNA) and BIS sensitivity mediated the association between PLES and NA (effect = 0.042; SE = 0.018; 95%CIs (0.011; 0.080)). Greater PLES was associated with greater CATS neglect/negative atmosphere and higher scores on CATS neglect/negative atmosphere were associated with greater BIS sensitivity which, in turn, was positively associated with NA. CATS neglect/negative atmosphere was also positively associated with NA but the association between PLES and NA was not significant. Jointly, PLES, CATS NAA, and BIS sensitivity accounted for 52% of the variance in NA (Table 3). Indirect effect 1 was (effect = 0.096; SE = 0.042; 95%CIs (0.017; 0.180)), but Indirect effect 2 was not (effect = 0.029; SE = 0.014; 95%CIs (0.007; 0.062)) supported.

**Table 3:** Model coefficients for serial mediation models testing effects of prenatal maternal stress on the prenatal life events scale (PLES) through childhood neglect/negative atmosphere and adolescent BIS sensitivity to adolescent affective outcomes

	Consequent								
	*M*_*1*_ (CATS NNA)			*M*_*2*_ (BIS)			*Y* (NA)		
Antecedent	*B*	*b*	SE	*B*	*b*	SE	*B*	*b*	SE
*X* (PLES)	0.143	0.934*	0.380	0.041	0.422	0.575	0.024	0.126	0.237
*M*_*1*_ (CATS NNA)	–	–	–	0.503	0.789***	0.091	0.205	0.163***	0.045
*M*_*2*_ (BIS)	–	–	–	–	–	–	0.588	0.298***	0.026
Constant	–	9.453***	0.619	–	43.034***	1.116	–	− 1.829^§^	1.074
	*R*^*2*^ = 0.02, *F*(1, 266) = 6.022*			*R*^*2*^ = 0.26, *F*(2, 265) = 40.851***			*R*^*2*^ = 0.52, *F*(3, 264) = 100.479***		

	*M*_*1*_ (CATS NNA)			*M*_*2*_ (BIS)			*Y* (ED)		
Antecedent	*B*	*b*	SE	*B*	*b*	SE	*B*	*b*	SE
*X* (PLES)	0.143	0.934*	0.380	0.041	0.422	0.575	0.043	0.712	0.791
*M*_*1*_ (CATS NNA)	–	–	–	0.503	0.789***	0.091	0.195	0.492***	0.145
*M*_*2*_ (BIS)	–	–	–	–	–	–	0.598	0.962***	0.083
Constant	–	9.453***	0.619	–	43.034***	1.116	–	23.890***	3.386
	*R*^*2*^ = 0.02, *F*(1, 266) = 6.021*			*R*^*2*^ = 0.36, *F*(2, 265) = 40.851***			*R*^*2*^ = 0.52, *F*(3, 264) = 102.234***		

	*M*_*1*_ (CATS NNA)			*M*_*2*_ (BIS)			*Y* (aggression)		
Antecedent	*B*	*b*	SE	*B*	*b*	SE	*B*	*b*	SE
*X* (PLES)	0.140	0.943*408		0.051	0.538	0.612	− 0.053	− 0.695	0.789
*M*_*1*_ (CATS NNA)	–	–	–	0.506	0.799***	0.093	0.239	0.471*	0.197
*M*_*2*_ (BIS)	–	–	–	–	–	–	0.238	0.297***	0.105
Constant	–	9.401***	0.626	–	42.897***	1.131	–	41.038***	4.648
	*R*^*2*^ = 0.2 *F*(1, 266) = 5.351*			*R*^*2*^ = 0.27, *F*(2, 265) = 40.602***			*R*^*2*^ = 0.17, *F*(3, 264) = 12.675***		

*B* standardized regression coefficients, *b* unstandardized coefficients, *SE* heteroscedasticity-consistent standard error estimator

***p < 0.001; **p < 0.01; *p < 0.05; ^§^0.1 > *p* < 0/05

#### Emotion Dysregulation as the Outcome

CATS neglect/negative atmosphere and BIS sensitivity mediated the association between PLES and DERS (effect = 0.043; SE = 0.017; 95%CIs (0.012; 0.079)). Greater PLES was associated with greater CATS neglect/negative atmosphere and higher scores on CATS neglect/negative atmosphere was associated with greater BIS sensitivity which, in turn, was positively associated with greater ED. CATS neglect/negative atmosphere was also associated with greater ED but the associations between PLES and BIS and PLES and ED were not significant. Jointly, PLES, CATS neglect/negative atmosphere, and BIS sensitivity accounted for 52% of the variance in ED (Table 3). Indirect effect 1 was (effect = 0.028; SE = 0.014; 95%CIs (0.006; 0.060)), but Indirect effect 2 was not (effect = 0.025; SE = 0.033; 95%CIs (− 0.038; 0.095)) supported.

#### Aggression as the Outcome

CATS neglect/negative atmosphere and BIS sensitivity mediated the association between PLES and aggression (effect = 0.017; SE = 0.010; 95%CIs (0.002; 0.042)). Greater PLES was associated with greater CATS neglect/negative atmosphere and higher scores on CATS neglect/negative atmosphere was associated with greater BIS sensitivity which, in turn, was positively associated with aggression. CATS neglect/negative atmosphere was also positively associated with aggression but the associations between PLES and BIS and PLES and aggression were not significant. Jointly, PLES, CATS neglect/negative atmosphere, and BIS sensitivity accounted for 17% of the variance in aggression (Table 3). Indirect effect 1 was (effect = 0.034; SE = 0.020; 95%CIs (0.004; 0.081)), but Indirect effect 2 was not (effect = 0.012; SE = 0.014; 95%CIs (− 0.016; 0.039)) supported.

### Mediation Analyses with the PSS as the Predictor

#### Negative Affectivity as the Outcome

CATS neglect/negative atmosphere and BIS sensitivity mediated the association between PSS and NA (effect = 0.070; SE = 0.020; 95%CIs (0.031; 0.112)). Greater PSS was associated with greater CATS neglect/negative atmosphere and CATS neglect/negative atmosphere was positively associated with BIS sensitivity (the association between PSS and BIS sensitivity was not significant) which, in turn, was positively associated with NA (the association between PSS and NA was not significant but CATS neglect/negative atmosphere was positively associated with NA). Jointly, PSS, CATS neglect/negative atmosphere, and BIS sensitivity accounted for 52% of the variance in NA (Table 4). Indirect effect 1 was (effect = 0.049; SE = 0.020; 95%CIs (0.016; 0.091)) but Indirect effect 2 was not (effect = 0.024; SE = 0.032; 95%CIs (− 0.036; 0.088)) supported.

**Table 4:** Model coefficients for serial mediation models testing effects of prenatal maternal stress on the perceived stress scale (PSS) through childhood neglect/negative atmosphere and adolescent BIS sensitivity to adolescent affective outcomes

	Consequent								
	*M*_*1*_ (CATS NNA)			*M*_*2*_ (BIS)			*Y* (NA)		
Antecedent	*B*	*b*	SE	*B*	*b*	SE	*B*	*b*	SE
*X* (PSS)	0.242	0.224***	0.058	0.041	0.060	0.080	0.034	0.026	0.035
*M*_*1*_ (CATS NNA)	–	–	–	0.496	0.780***	0.097	0.201	0.159***	0.047
*M*_*2*_ (BIS)	–	–	–	–	–	–	0.587	0.294***	0.026
Constant	–	3.262^§^	1.820	–	41.702***	2.388	–	1.376	1.395
	*R*^*2*^ = 0.24, *F*(1, 262) = 15.038***			*R*^*2*^ = 0.28, *F*(2, 261) = 41.879***			*R*^*2*^ = 0.52, *F*(3, 260) = 93.879***		

	*M*_*1*_ (CATS NNA)			*M*_*2*_ (BIS)			*Y* (ED)		
Antecedent	*B*	*b*	SE	*B*	*b*	SE	*B*	*b*	SE
*X* (PSS)	0.242	0.224***	0.058	0.041	0.060	0.080	0.125	0.292*	0.118
*M*_*1*_ (CATS NNA)	–	–	–	0.496	0.780***	0.097	0.169	0.426**	0.147
*M*_*2*_ (BIS)	–	–	–	–	–	–	0.598	0.959***	0.080
Constant	–	3.262^§^	1.820	–	41.702***	2.388	–	15.974***	4.480
	*R*^*2*^ = 0.06, *F*(1, 262) = 15.038***			*R*^*2*^ = 0.26, *F*(2, 261) = 41.879***			*R*^*2*^ = 0.54, *F*(3, 260) = 101.171***		

	*M*_*1*_ (CATS NNA)			*M*_*2*_ (BIS)			*Y* (aggression)		
Antecedent	*B*	*b*	SE	*B*	*b*	SE	*B*	*b*	SE
*X* (PSS)	0.238	0.224***	0.060	0.054	0.081	0.081	− 0.081	− 0.150	0.113
*M*_*1*_ (CATS NNA)	–	–	–	0.497	0.786**	0.100	0.249	0.491*	0.200
*M*_*2*_ (BIS)	–	–	–	–	–	–	0.237	0.296**	0.106
Constant	–	3.243^§^	1.860	–	41.039***	2.420	–	45.060***	5.602
	*R*^*2*^ = 0.06, *F*(1, 262) = 14.131***			*R*^*2*^ = 0.26, *F*(2, 261) = 42.219***			*R*^*2*^ = 0.16, *F*(3, 260) = 12.504***		

*B* standardized regression coefficients, *b* unstandardized coefficients, *SE* heteroscedasticity-consistent standard error estimator

***p < 0.001; **p < 0.01; *p < 0.05; ^§^0.1 > *p* < 0.05

#### Emotion Dysregulation as the Outcome

CATS neglect/negative atmosphere and BIS sensitivity mediated the association between PSS and DERS (effect = 0.072; SE = 0.021; 95%CIs (0.034; 0.114)). Greater PLES was associated with greater CATS neglect/negative atmosphere and higher scores on both were associated with greater BIS sensitivity which, in turn, was positively associated with ED (the associations between PLES and ED and CATS neglect/negative atmosphere and ED were not significant). Jointly, PLES, CATS neglect/negative atmosphere, and BIS sensitivity accounted for 54% of the variance in ED (Table 4). Indirect effect was 1 (effect = 0.041; SE = 0.018; 95%CIs (0.010; 0.081)) but Indirect effect 2 was not (effect = 0.024; SE = 0.032; 95%CIs (−0.035; 0.089)) supported.

#### Aggression as the Outcome

CATS neglect/negative atmosphere and BIS sensitivity mediated the association between PSS and DERS (effect = 0.028; SE = 0.015; 95%CIs (0.006; 0.062)). Greater PSS was associated with greater CATS neglect/negative atmosphere and higher scores on CATS neglect/negative atmosphere were associated with greater BIS sensitivity (the association between PSS and BIS sensitivity was not significant) which, in turn, was positively associated with aggression. CATS neglect/negative atmosphere was also positively associated with aggression but the associations between PSS and aggression was not significant). Jointly, PSS, CATS neglect/negative atmosphere, and BIS sensitivity accounted for 17% of the variance in aggression (Table 4). Indirect effect 1 was (effect = 0.059; SE = 0.025; 95%CIs (0.016; 0.112)), but Indirect effect 2 was not (effect = 0.013; SE = 0.014; 95%CIs (− 0.013; 0.041)) supported.

**Fig. 1:**
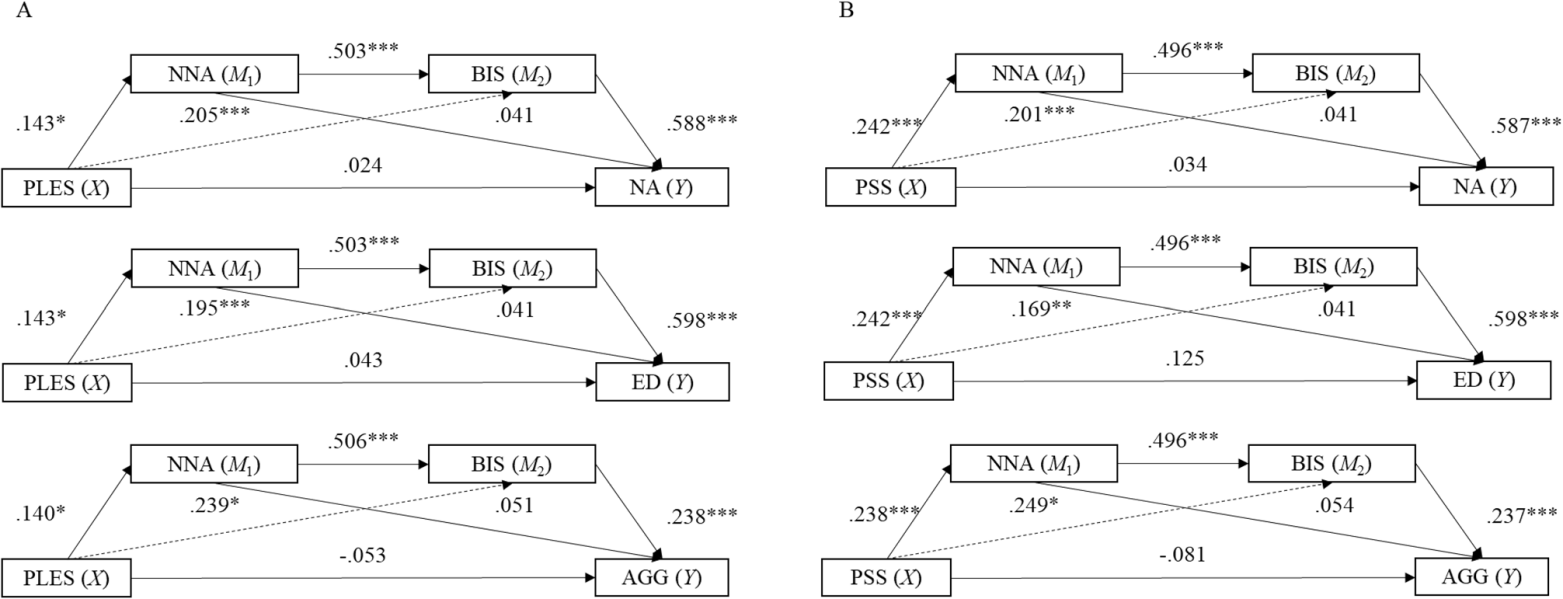
Visual summary of serial mediation results with childhood neglect/ negative atmosphere (NNA) and adolescent behavioral inhibition system (BIS) sensitivity operating in serial and mediating the association between prenatal maternal stress and adolescent affective outcomes, with **A** the prenatal life events scale (PLES) and **B** the perceived stress scale (PSS) as indices of prenatal stress. Solid lines indicate models with indirect effects supported (indirect effects 1 and 3, across models) and dashed lines indicate models with indirect effects not supported (indirect effect 2, across models). Coefficients are standardized regression weights. NA negative affectivity, ED emotion dysregulation, AGG aggression. ***p < 0.001; **p < 0.01; *p < 0.05; 0.1 > *p *< 0.05

For a visual summary of mediation results, see Fig. 1.

### Follow-up Moderated Mediation Analyses

Adolescent ADHD status did not moderate the direct or indirect effect (through CATS neglect/negative atmosphere**)** of PLES on BIS (index = − 1.205; SE = 0.702; 95%CIs (− 2.599; 0.153)), on NA (index = − 0.592; SE = 0.360; 95%CIs (− 1.326; 0.114)), on ED (index = 1.855; SE = 1.124; 95%CIs (− 4.134; 0.404)), or on aggression (index = 1.007; SE = 0.700; 95%CIs [− 2.256; 0.567)). Maternal internalizing symptoms also did not moderate the direct or indirect effect (through CATS neglect/negative atmosphere) of PSS on BIS (index = − 0.081; SE = 0.106; 95%CIs (−0.287; 0.130)) on NA (index = − 0.040; SE = 0.053; 95%CIs (−0.143; 0.071)), on ED (index = − 0.040; SE = 0.109; 95%CIs (−0.261; 0.177)), or on aggression (index = − 0.040; SE = 0.109; 95%CIs (− 0.261; 0.177)).

Maternal internalizing symptoms did not moderate the direct or indirect effect (through CATS neglect/negative atmosphere**)** of PLES on BIS (index = − 0.042; SE = 0.032; 95%CIs (− 0.112; 0.017)), on NA (index = − 0.021; SE = 0.017; 95%CIs (− 0.060; 0.008)), on ED (index = − 0.065; SE = 0.052; 95%CIs (− 0.182; 0.025)), or on aggression (index = − 0.025; SE = 0.041; 95%CIs (− 0.111; 0.052)). Maternal internalizing symptoms also did not moderate the direct or indirect effect (through CATS neglect/negative atmosphere) of PSS on BIS (index = − 0.006; SE = 0.004; 95%CIs (− 0.014; 0.001)) on NA (index = − 0.003; SE = 0.002; 95%CIs (− 0.007; 0.001)), on ED (index = − 0.009; SE = 0.006; 95%CIs (− 0.021; 0.002)), or on aggression (index = − 0.004; SE = 0.004; 95%CIs (− 0.014;0.004)).

Adolescent sex status did not moderate the direct or indirect effect (through CATS neglect/negative atmosphere (NNA)**)** of PLES on BIS (index = 0.677; SE = 0.653; 95%CIs (− 0.528; 2.018)), on NA (index = 0.373; SE = 0.369; 95%CIs (− 0.309; 1.146)), on ED (index = 1.150; SE = 1.159; 95%CIs (− 1.001;3.560)), or on aggression (index = 1.145; SE = 0.852; 95%CIs (− 0.317; 3.002)). Adolescent sex also did not moderate the direct or indirect effect (through CATS neglect/negative atmosphere (NNA)) of PSS on BIS (index = 0.068; SE = 0.085; 95%CIs (− 0.096; 0.242)), on NA (index = 0.037; SE = 0.047; 95%CIs (− 0.052; 0.136)), on ED (index = 0.109; SE = 0.138; 95%CIs (− 0.140; 0.400)), or on aggression (index = 0.101; SE = 0.100; 95%CIs (− 0.087; 0.295)).

## Discussion

Our goals in this study were to assess, in middle-late adolescents, whether prenatal maternal stress—conceptualized and measured as both relatively minor, daily stressors and as relatively major, life events stressors—is associated with indices of affective processing, specifically, affectivity, aggression, and emotion dysregulation and whether these associations are mediated, serially, by differences in the childhood home environment and adolescent BIS sensitivity. We also examined whether adolescent ADHD diagnosis or maternal internalizing symptoms moderated any of the mediational effects.

This study is one of a handful of examinations of transdiagnostic outcomes of prenatal maternal stress, with findings applicable across the general and clinical populations. To the best of our knowledge, this was the first evaluation of a serial mediational effect and of the mediational effect of adolescent BIS sensitivity.

Findings of bivariate correlation analyses were generally as expected, with a positive association of PLES scores with BIS sensitivity [110] and with NA [110, 111]. These findings are not only consistent with earlier results, but extend those to the transdiagnostic characteristics of negative affectivity, aggression, and emotion dysregulation, in a relatively understudied developmental group, middle-late adolescents. Interestingly, there was a differential pattern, where prenatal maternal stress caused by major life events was associated with all measured adolescent outcomes but ED whereas prenatal maternal stress caused by minor daily stressors was not associated with any of the adolescent outcomes but ED. All of these relations corresponded to small effects, perhaps because several third variables influence such relations (including the ones assessed here) and/ or because prenatal maternal stress was mother-reported whereas adolescent outcomes were self-reported.

Also as expected, the hypothesized mediators, CATS neglect/negative atmosphere and BIS sensitivity were positively correlated with each other [85] and also with all assessed outcomes [40, 112–116]. These relations corresponded to medium to large effects, likely driven by their relative phenomenological and temporal proximity and/ or partly due to shared method variance. In line with what is known about age and sex differences, older age was associated with greater BIS sensitivity [117, 118] and girls reported greater BIS sensitivity. Others have previously found a curvilinear relation between age and aggression, where aggression increased until early-middle adolescence and declined by late adolescence [119, 120] and no sex-difference with regard to emotional neglect and abuse [121] whereas here, we wound older age was associated with greater aggression and girls reported greater CATS neglect/negative atmosphere. Perhaps most importantly, prenatal maternal stress caused by major life events was positively associated with prenatal maternal stress caused by minor daily stressors but with the correlation coefficient corresponded to a small effect, indicating that although the two variables are associated, they are not isomorphic (or redundant) and thus represent related but distinct phenomena.

When examined in greater complexity, i.e., in a serial mediation model, both indices of prenatal maternal stress were associated with adolescent affective outcomes, through the serial effects of differences in the early childhood home atmosphere/environment and in adolescent BIS sensitivity. Of import, the effect of prenatal stress on the herein assessed adolescent affective outcomes was only significant in the presence of a mediational effect of childhood neglect/ negative atmosphere and BIS sensitivity (i.e., full mediation). The mediational effect of childhood neglect/ negative atmosphere (indirect effect 1) was significant even in the absence of the subsequent mediational effect of BIS sensitivity. This is consistent with earlier findings indicating an association between prenatal maternal stress and subsequent difficulties with parenting [122] and child maltreatment [71]. Our finding further extends these prior results insofar as it suggests an effect of these variables on adolescent affective outcomes. The mediational effect of BIS sensitivity (indirect effect 2) was only significant in the presence of a preceding mediational effect of childhood neglect/ negative atmosphere. This set of results have both conceptual and clinical implications.

Regarding conceptual implications, *first*, these findings underscore the importance of examining the effect of mechanisms—when examined in the context of mediators, the direct path between prenatal maternal stress and outcomes was not significant, indicating the observed relation was fully mediated by the assessed mechanisms. This finding is consistent with contemporary thinking about the complexity of relations between variables indexing human development and psychology; that accounting for relevant mechanisms and influences may reveal small or otherwise undetected effects [123].

*Second*, as noted, although the mechanisms via which gestational stress is associated with neurodevelopment and increases risk for offspring behavioral problems have yet to be elucidated [19], a leading hypothesis is that prenatal maternal stress impacts, via fetal exposure to elevated cortisol, offspring brain systems related to stress processing and regulation, such as the septohippocampal system. Our results are consistent with this hypothesis insofar as they show that prenatal maternal stress is associated with adolescent offspring affective problems through greater BIS sensitivity, with the septohippocampal system, and its monoaminergic brainstem afferents hypothesized to be implicated in BIS sensitivity [124].

Regarding clinical implications, establishing that an early-emerging characteristic may be a protective or risk factor for later functional impairments, behavioral problems, or psychopathology enhances conceptualization of developmental trajectories to such outcomes. Arguably, however, the practical significance of such a characteristic and its role is closely linked to the degree to which it is a promising intervention—prevention or treatment—target and that is largely determined by the degree to which it is malleable [125]. It is against this backdrop that we discuss the relevance of the current set of results for prevention of adolescent affective problems such as negative affectivity, aggression, and ED and the sequalea of such problems, including diagnosable psychopathology [126–128]. In the context of the variables examined here, the first point of intervention would need to be during the prenatal period and focus on decreasing maternal stress. Such preventions and treatments are available and include cognitive-behavioral, interpersonal, and psychoeducational strategies, with data indicating short-term efficacy of cognitive and psychoeducational techniques for reducing prenatal maternal stress [129–131]. Early evidence shows Both mindfulness-based interventions and yoga may also effectively decrease prenatal maternal stress, but methodologically rigorous studies are needed to confirm observed effects [131–134]. Virtual reality, although also promising, is less available than these approaches and more research is needed to establish its efficacy [135]. In case of children for whom prenatal maternal stress had already occurred (e.g., who present to clinical care during early childhood or later), consistent with a differential susceptibility framework, an alternative point of intervention would be during childhood and focus on decreasing neglect/ negative atmosphere and improving the early childhood home atmosphere/environment (e.g., through parent training; [136]. In terms of scientific rigor, studies on parenting programs are heterogeneous, with some methodologically sound designs assessing efficacy of e.g., Group Family Nurse Partnership [137], REAL Fathers Initiative [138], and SOS Help for Parents [139]. In case of children for whom both the prenatal maternal stress and the negative childhood home atmosphere/environment had occurred (e.g., who present to clinical care during early adolescence or later), there is yet an alternative point and focus of intervention. In such cases, intervention would be indicated during adolescence and to aim at attenuating BIS sensitivity. Data show the BIS may be malleable as environmental (vs. genetic) effects contribute to changes in BIS sensitivity [140]. Although to our knowledge, no interventions explicitly target BIS sensitivity, certain interventions target and have been observed to alter reinforcement sensitivity (e.g., Cognitive Remediation Therapy targets punishment sensitivity and Transcranial Magnetic Stimulation targets reward sensitivity) [141, 142]. Other interventions do not target reinforcement sensitivity but have been shown to reduce BIS sensitivity (e.g., Behavioral Activation Therapy and certain mindfulness types) [143, 144]. Pharmacological treatments, e.g., anxiolytic drugs also reduce BIS sensitivity [36]. Of note, BIS sensitivity is not to be conflated with anxiety. There is certainly overlap between BIS hyperactivity and excessive anxiety, as BIS hyperactivity may result in excessive focus on environmental threat and, as an *indirect* consequence, on threatening associations with previous stimuli [36]. Nonclinical and clinically anxious individuals exhibit an automatic, preferential attention to threat (i.e., attentional bias) [145]. Despite this overlap, the response of the defense system of which the BIS is part is *context- or state-dependent*. In contrast, individuals with elevated levels of anxiety *stably* perceive the environment as dangerous; as such, elevated trait anxiety is associated with negative schema, hyper-vigilance to threatening information, and at the memory level, hyper-recall of threatening information [146].

### Limitations and Future Directions

Prenatal maternal stress was assessed via retrospective report, which is associated with potential biases, including, but not limited to biases resulting from current psychological disorders or functioning. Three lines of evidence lend credence to the validity of assessing prenatal maternal stress via retrospective report.

First, although past 6 months maternal internalizing symptoms were positively associated with both measures of prenatal maternal stress (prenatal stress caused by major life events and prenatal stress caused by minor daily stressors) and these two measures of prenatal stress were also positively associated with each other, the magnitude of these relations were mostly small. This arguably indicates, on one hand, that current maternal internalizing symptoms did not to a confounding extent influence maternal report of prenatal stress. Further, that mothers differentiated between the two types of stressors suggests absence of a systematic bias.

Second, earlier data evince the accuracy of retrospective report of relevant characteristics and information, such as descriptions of the family environment 25 years ago validated against prospective measures of such environment (moderate correlations of 0.30–0.45 [147]; maternal report of breastfeeding history from 20 years ago validated against clinic charts (strong correlations of 0.86) [148]; maternal report about a range of pre and peri-natal factors (e.g., infant birth weight, infant admission to special care baby unit, method of delivery, smoking during pregnancy, high blood pressure/oedema during pregnancy) from four to nine years ago validated against antenatal records [149] and against maternal report 6 months postpartum [150]; or parental report of child birth information from 12 years ago validated against hospital records [151].

Third, we repeated our analyses conducted with the “mean prenatal life events distress score” using “the number of prenatal life events” as the PLES predictor and replicated our findings (see Supplement). This is meaningful as the former score comprises items assessing the extent to which one was negatively affected by events that occurred whereas the latter comprises items assessing whether or not such events occurred. Arguably, the former is considerably more subject to recall bias than the latter. Although these data lend credence to the herein obtained retrospective report of prenatal stress, a recall bias cannot be completely ruled out. As such, our data are best conceptualized as preliminary indication that the herein observed relations are worthy of examination in considerably more resource-consuming longitudinal, prospective designs.

Given poor internal consistency of the other CATS subscales, only one aspect of the early childhood home atmosphere/environment was examined in our research (i.e., neglect/negative atmosphere), future research may examine whether our findings generalize to other aspects of the childhood home environment, such as physical abuse and neglect or sexual abuse. Similarly, only one aspect of reinforcement sensitivity was tested in this study (i.e., BIS sensitivity). Evidence shows prenatal maternal nutrition and stress affect offspring mesocorticolimbic system [152], and such prenatal programming of offspring mesocorticolimbic system appears to play a role in the development of traits related to behavioral activation system (BAS) sensitivity, such as attention toward salient stimuli, reward sensitivity as well as extraversion, novelty seeking, and impulsivity [153]. Accordingly, future research may examine whether the herein obtained results generalize to other aspects of reinforcement sensitivity, such as BAS sensitivity.

Mediation can be demonstrated in cross-sectional research [154], but only via statistical criteria [123, 155–158]. Accordingly, we established atemporal mediation, mathematically/ statistically. As we tested but did not find support for reversed models—as recommended e.g., in cross-sectional designs, where temporal precedence is not definitively established [159–163], our findings evince unidirectionality of observed effects and indicate additional research is warranted, e.g., experimental or prospective studies to establish temporal mediation and thus causation [161, 164].

We examined relations across a set of a priori chosen variables, selected based on theory and prior empirical data. Certainly, the relations between prenatal maternal stress and adolescent outcomes is complex, with several additional variables also influencing such relations. Although we aimed to assess some of these variables, including maternal smoking or drug use during pregnancy, only a small number of mothers reported they smoked or used alcohol or drugs, indicating there may be need in the future to oversample mothers who engage in substance use. Examples of pertinent variables not assessed here include child genetic predisposition or life events, and parental emotion regulation or parenting style. In support, there is evidence that prenatal maternal stress may have greatest impact on areas of functioning in which the offspring has a genetic vulnerability; in one study, prenatal maternal anxiety was associated with adolescent offspring ADHD outcome at age 15 years only in children with a specific variation of the COMT gene [60].

In intending to assess the moderational effect of maternal internalizing symptomology, we assessed maternal anxiety and depressive symptoms as experienced during the past 6 months. Current and past 6 months internalizing symptoms may not be related to maternal internalizing symptomology during pregnancy or after, however, it is a good proxy for the likelihood of maternal anxiety or depression as well as for genetic predisposition to offspring psychopathology [87]. Nevertheless, to be able to make stronger claims about the moderational effect of maternal psychopathology, it will be important to collect data on this variable during and after pregnancy.

As noted, we found outcomes were differentially associated with prenatal maternal stress caused by major life events vs. daily stressors; the former was associated NA and aggression whereas the latter was only associated with ED. It would be premature to speculate on this pattern based on the current findings, but whether these differential effects replicate (and what might be driving them) may be conceptually relevant to explore in the future.

We defined adolescence based on chronological age without accounting for biological age or pubertal status, though these may not necessarily correspond. It thus remains an outstanding question whether these findings replicate if adolescence is defined based on biological maturity.

Finally, the current sample was a community-based sample, with the majority of youth White. It will be important to examine whether results generalize to clinical populations, ethnic minorities and other racial groups as well as to collect data on symptoms, beyond children and parents, from teachers. Related, as described in *Methods,* adolescents were oversampled for ADHD. As such, sampling was not random and findings may not correspond to what would be observed in a randomly selected group of adolescents. Although we assessed and did not find evidence for a moderational effect of ADHD status, there are associated features of ADHD (e.g., at the group level, differences in IQ or SES) that were not evaluated in this manner but may have a bearing on the results.

## Summary

In this first study assessing the serial mediational effect of differences in the childhood home environment and adolescent behavioral inhibition system sensitivity on the association between prenatal maternal stress and adolescent affective outcomes, we found that the relation between prenatal maternal stress and adolescent affective outcomes is mediated by both childhood neglect/negative atmosphere and adolescent behavioral inhibition system sensitivity. These relations held across types of maternal stress, i.e., across stress caused by major, life events stressors and stress caused by minor, daily stressors and were not moderated by adolescent ADHD diagnosis or by current/recent maternal internalizing symptomology. On its own, neither childhood neglect/negative atmosphere nor adolescent behavioral inhibition system sensitivity exerted a mediational effect, indicating it is in the modeled and tested serial order that they account for the relation between prenatal maternal stress and adolescent negative affectivity, aggression, and ED. Prevention and intervention efforts aimed at reducing negative adolescent affective outcomes and the potential psychiatric sequelae of such outcomes are to be tailored, with regard to their focus and target, to the developmental phase during which the intervention occurs.

### Supplementary Information

Below is the link to the electronic supplementary material.

Supplementary file1 (DOCX 70 kb)

## Data Availability

The datasets generated and/or analyzed during the current study are available from the corresponding author on reasonable request.

## Footnotes

1 Mainly as maternal psychiatric symptoms, such as anxiety and depression, major life events, military invasion, natural disasters.

2 The macros provide a 95% CI around the indirect effect. When zero is not in the 95% CI, the indirect effect is different from zero at *p* < 0.05 (two tailed).
