# Supplementary material for: The Association Between Prenatal Maternal Stress and Adolescent Affective Outcomes is Mediated by Childhood Maltreatment and Adolescent Behavioral Inhibition System Sensitivity
Source: Child Psychiatry Hum Dev. 2023 Feb 4;55(5):1–21. doi: 10.1007/s10578-023-01499-9 (PMC11362206; doi:10.1007/s10578-023-01499-9)
Supplement: Supplementary file 1 — Supplementary file1 (DOCX 70 kb) [file 10578_2023_1499_MOESM1_ESM.docx]

**SUPPLEMENTARY INFORMATION TO:**

**PRENATAL MATERNAL STRESS AND ADOLESCENT AFFECT: CHILDHOOD MALTREATMENT AND ADOLESCENT BIS SENSITIVITY AS MECHANISMS**

BLINDED

**Method**

**Measures**

***Clinician administered measures***

***Wechsler Intelligence Scales.*** To estimate cognitive ability, abbreviated versions of the Wechsler Intelligence Scales were used. The Wechsler Intelligence Scale for Children, Fourth Edition [1] for youth under the age of 17 and the Wechsler Adult Intelligence Scale – Fourth Edition [2] for youth 17 and above. Two subtests were administered of the Perceptual Reasoning subscales (PR), Matrix Reasoning and Picture Concepts (WISC), and Matrix Reasoning and Visual Puzzles (WAIS). Two subtests were administered of the Verbal Comprehension subscales (VC), Similarities and Vocabulary (both WISC and WAIS). These subtests allow for calculation of a Perceptual Reasoning Index (PRI) and Verbal Comprehension Index (VCI) estimate and percentile ranks corresponding to these estimates were used as indices of cognitive ability.

***Adolescent self-report measures***

***Child Abuse and Trauma Scale (CATS)*** [3]***.*** The CATS is a 38-item self-report measure of stress and/ or trauma in the respondents’ home during their childhood, comprised of 3 subscales: *Neglect/Negative Home Atmosphere* (NNA; 14 items assessing loneliness and general stress at home, e.g., *“Were your parents unhappy with each other?”*)*,* Punishment (6 items assessing severe, strict, or irrational punishment, e.g., “*Were you expected to follow a strict code of behavior in your home?*”) and *Sexual Abuse* (6 items assessing direct and indirect sexual abuse, e.g., *“Did you ever witness the sexual mistreatment of another family member?”*). Respondents rate the frequency with which they experienced each event on a 5-point Likert-type response format scale (0 – ‘Never’ to 4 – ‘Always’), basing their ratings on the behavior of whichever parent executed the described behavior more frequently/ severely [3]. Although originally designed for use with adults, the CATS is appropriate for use with adolescents (Sanders, 1995). Prior findings indicate the CATS has acceptable psychometric properties, with Cronbach’s alpha values as follows: .86 for NNA, .76 for Sexual Abuse, .63 for Punishment, and .90 for Total CATS (Sanders, 1995). The Hungarian translation [4] demonstrated poor to excellent internal consistency, with alphas ranging from .54 to .90 for the three subscales and Total CATS.

In the current sample, internal consistency of the subscales ranged from unacceptable to good, with Cronbach’s alpha as follows: NNA=.843; Punishment=.621; Sexual Abuse=.519, and Total CATS=.893. In the current study, data from the NNA subscale were analyzed.

***Reinforcement Sensitivity Theory of Personality Questionnaire (RST-PQ)***^[[1]](#footnote-1)^ [5]***.*** The RST-PQ is a 79-item self-report measure of the revised Reinforcement Sensitivity Theory (rRST) personality dimensions comprising three subscales: Flight-Fight-Freeze system (FFFS; 10 items, e.g., *I would be frozen to the spot by the sight of a snake or spider, There are some things that I simply cannot go near*), Behavioral Activation System (BAS; 32 items), and Behavioral Inhibition System (BIS; 23 items, e.g., *When nervous, I find it hard to say the right words, I take a long time to make decisions*), and two additional subscales developed to complement the core RST-PQ: Defensive Fight (8 items, e.g., *I can be an aggressive person when I need to be*) and Panic (6 items, e.g., *I sometimes wake up in a state of terror*). Prior findings suggest Defensive Fight, as it loads highly on BAS and Panic, as it loads highly on both FFFS and BIS, should nevertheless be considered separately from the three main subscales ([5]. Of interest to the current study, the BAS subscale consists of four further subscales: Reward Interest (7 items, e.g., *I regularly try new activities just to see if I enjoy them*), Goal-Drive Persistence (7 items, e.g., *I* *often overcome hurdles to achieve my ambitions*), Reward Reactivity (10 items, e.g., *Sometimes even little things in life can give me great pleasure*) and Impulsivity (8 items, e.g., *I find myself doing things on the spur of the moment*). Respondents rate how accurately each item describes them on a four-point Likert-type response format scale (1 – ‘not at all’ to 4 – ‘highly’). Higher scores indicate higher sensitivity to reinforcement. Prior findings indicate that RST-PQ demonstrated good internal consistency and adequate convergent and discriminant validity with other personality measures (e.g., Eysenck Personality Questionnaire-Revised [EPQ-R], BIS/BAS, State-Trait Anxiety Inventory [STAI]) [5]–[7].

For purposes of the current study, the English version of the RST-PQ was translated into Hungarian following evidence-based guidelines: (1) the English version was translated into Hungarian by three independent translators; (2) these three translations were combined into a single “summary translated” measure by a fourth independent translator, reconciling all discrepancies across the three translations/ors; (3) the “summary” was back-translated into English by two additional independent translators and (4) the two back-translations were combined into a single “summary back-translated” measure by members of the research team, reconciling all discrepancies in a manner that the “summary back-translation” measure best matches the Hungarian “summary translated” measure. This “summary back-translated” questionnaire was sent to the original author(s) who provided the research team with feedback and ultimately approved the translated measure (P. Corr, personal communication, May 29, 2019).

In the current sample, internal consistency of the subscales ranged from acceptable to excellent, with Cronbach’s alpha values as follows: BAS=.886; BIS=.912; FFFS=.763; Panic=.794; Defensive Fight=.786; Total RSTP-Q=.900. In the current study, data from the BIS subscale were analyzed.

***Positive and Negative Affect Schedule (PANAS)*** [8]***.*** The PANAS is a 20-item self-report measure of state and/or trait positive and negative affect, comprised of two subscales, the positive affect (PA) subscale, reflecting the extent to which a person feels enthusiastic, active and alert, and a negative affect (NA) subscale, reflecting a general dimension of subjective distress and a variety of aversive mood states such as anger, contempt, disgust, fear, guilt, and nervousness. Respondents rate the extent to which they are experiencing each mood state “right now” (i.e., state version) or “during the past two weeks” (i.e., trait version) on a five-point Likert-type response format scale (1 – ‘very slightly or not at all’ to 5 – ‘very much’). Higher scores on the PA and NA subscales indicate greater positive and negative affect, respectively. Prior findings indicate that PANAS scales have good internal consistency (αs ranging from .86 to .90 for PA and from .84 to .87 for NA) and good convergent and discriminant associations with distress and psychopathology measures of the underlying affectivity factors (e.g., Beck Depression Inventory [BDI], Hopkins Symptom Checklist [HSCL], STAI) [9]. The Hungarian translation also demonstrated acceptable psychometric properties, including good internal consistency (PA α=.82, NA α=.83 [alpha values are provided only to the second decimal in the source article]) [10].

In the current sample, the PANAS-trait was administered internal consistency of the subscales was good, with Cronbach’s alpha values as follows: NA=.848; PA=.823. In the current study, data from the PA and NA subscales were analyzed.

***Difficulties in Emotion Regulation Scale (DERS)*** [11]*.* The DERS is a 36 item self-report measure of emotion dysregulation (ED), comprised of six subscales, Nonacceptance of Emotional Responses (Nonacceptance, e.g., *When I’m upset, I become angry with myself for feeling that way*), Difficulties Engaging in Goal-Directed Behavior (Goals, e.g., *When I’m upset, I have difficulty concentrating*), Impulse Control Difficulties (Impulse, e.g., *When I’m upset, I become out of control*), Lack of Emotional Awareness (Awareness, e.g., *When I’m upset, I acknowledge my emotions*), Limited Access to Emotion Regulation Strategies (Strategies, e.g., *When I’m upset, I believe that wallowing in it is all I can do*), and Lack of Emotional Clarity (Clarity, e.g., *I have difficulty making sense out of my feelings*). Items are rated on a five-point Likert-type response format scale (1 – ‘Almost Never’ to 5 – ‘Almost Always’), with higher scores indicating greater difficulty with emotion regulation. Prior findings indicate the DERS has acceptable psychometric properties, including good internal consistency, good test–retest reliability, and adequate construct and predictive validity in multiple adolescent samples [12]–[16]. In addition, the DERS exhibited robust correlations with psychological problems reflecting ED [14] and physiological measures of ED [13]. The Hungarian translation also demonstrated acceptable psychometric properties, including good internal consistency (all αs>.70) as well as construct and convergent validity with the Zung Self-rated Depression Scale [17].

In the current sample, internal consistency of the subscales was acceptable to excellent, with Cronbach’s alpha values as follows: Awareness=.755; Clarity=.807; Goals=.863; Impulse=.862; Non-Acceptance=.835; Strategies=.861; Total DERS: .922. In the current study, data from the Total score were analyzed.

***Buss-Perry Aggression Questionnaire* *(BPAQ)*** [18]***.*** The BPAQ is a 29-item self-report measure of aggression, comprised of four subscales, anger (AN; 7 items), hostility (HS; 8 items), physical aggression (PA; 9 items), and verbal aggression (VA; 5 items). Items are rated on a five-point Likert-type response format scale (1 – ‘does not apply at all’ to 5 – ‘applies very well’), with higher scores indicating greater anger, hostility, and aggression. The measure is appropriate for use with adolescents 13-17 years of age [19]. Data indicate the BPAQ has adequate psychometric properties, including good internal consistency and test-retest reliability [18]. The BPAQ has been translated into Hungarian and confirmatory factor analyses generally indicated the original factor structure was replicated, with hostility items (with the exception of item 1, “*I am sometimes eaten up with jealousy*”) loading onto one factor, physical aggression items loading onto another factor, and verbal aggression items loading onto yet another factor. The anger subscale was only moderately replicated, as the majority of the original anger items loaded either onto an anger factor or onto the hostility factor [20]. Correlation coefficients between the *a priori* defined BPAQ subscale scores and the empirically derived factors were .92 for hostility, .86 for physical aggression, .86 for verbal aggression, and .63 for anger. The coefficients of congruence between factor loadings of the original BPAQ subscales, and those of the empirically derived factors were .39 for anger, .79 for hostility, .77 for physical aggression, and .58 for verbal aggression. Internal consistency estimates indicating good internal consistency for PA (α=.82), acceptable for HS (α=.75) and AN (α=.70) and questionable for VA (α=.68) [20].

In the current sample, internal consistency of the subscales was ≥acceptable, with Cronbach’s alpha values as follows: anger=.781; hostility=.773; physical aggression=.834; verbal aggression=.692; Total BPAQ=.901. In the current study, data from the Total score were analyzed.

***Parent-report measures***

***ADHD Rating Scale-5 (ARS 5)*** [21]***.*** The ARS-5 is a 30-item parent- and teacher-report measure of the past 6-month presence and severity of DSM-5 ADHD symptoms (9 inattentive symptom items and 9 hyperactivity/impulsivity symptom items) and functional impairment across six domains: relationship with significant others (family members for the home version), relationship with peers, academic functioning, behavioral functioning, homework performance and self-esteem (2x6 impairment items, with one set corresponding to inattention and one to hyperactivity/impulsivity). Parents and teachers rate items on a four-point scale ranging in case of symptoms from 0 (never or rarely) to 4 (very often) and in case of impairment from 0 **(“**no problem”) to 3 (“severe problem”), with higher scores indicating more severe symptoms and impairment. The ARS-5 is comprised of two symptoms scales, Inattention and Hyperactivity-Impulsivity, and a Total Scale. The ARS-5 is suitable for ages 5-17 years, with separate forms for children (5-10 years) and adolescents (11—17 years) and age-appropriate and DSM-5 compatible descriptions of symptoms. In the current study, the adolescent home (i.e., parent-report) version was used. The ARS-5 has well-established reliability of the adolescent, home version (e.g., internal consistency and 6-week test-retest reliability) and validity (i.e., factor structure; concurrent validity and predictive validity and clinical utility) [21].

For purposes of the current study, the English version of the ARS-5 was translated into Hungarian following the same procedures as for the RST-PQ. The original author approved the translated measure (G. DuPaul, personal communication, June 5, 2020). In the current sample, internal consistency of the ARS-5 was excellent, with a Cronbach’s alpha value of .944 for the Inattention subscale and .916 for the Hyperactivity/impulsivity subscale.

***Prenatal Life Event Scale (PLES)*** [22]. The PLES is a 29-item self-report measure assessing the occurrence of specific life events during pregnancy. In the first part of the questionnaire (18 items), mothers respond to a list of events (e.g., moving in with someone, getting robbed), first, by indicating whether or not they experienced the event during their pregnancy and second if they did experience it, they rate the degree to which the event negatively affected them on a 4-point Likert-type response format scale (0 – ‘not at all’ to 3 – ‘extremely’). In the second part of the questionnaire (10 items), mothers respond to a list of events by indicating whether or not either they or someone close to them experienced those during their pregnancy. Again, respondents rate the degree to which the event negatively affected them (regardless of whether the event happened to them or someone close to them), also specifying whether it was their own experience or someone else’s. Two indices of prenatal stress are obtained, one reflecting the number of prenatal life events and another, a mean distress score, reflecting the distress experienced as a result of such events [22]. Higher scores indicate a greater number of life events and greater stress as a result of such events.

For purposes of the current study, the English version of the PLES was translated into Hungarian following the same procedures as for the RST-PQ. The original author approved the translated measure (M. Lobel, personal communication, September 6, 2019). In the current sample, internal consistency of the mean prenatal life events distress score was acceptable, with a Cronbach’s alpha value of .706. In the current study, data were analyzed using the mean prenatal life events distress score (hereafter: PLES). (To increase confidence in obtained results, serial mediational analyses [see *Analytic Plan*] were repeated with the number of prenatal life events score. For corresponding findings, see Supplement.)

***Perceived Stress Scale (PSS)*** [23]***.*** The PSS is a 14-item self-report measure originally designed to assess everyday stress during a one-month period, but has since been adapted for use to assess maternal prenatal daily stress. The PSS does not have any subscales. A higher total score indicates greater stress [22]. Respondents rate, on a 5-point scale (‘Never’ to ‘Very often’) how often they felt a particular way, e.g., “*During your pregnancy, how often did you feel that you were unable to control the important things in your life?*”; “*During your pregnancy, how often did you feel on top of things?*” [24]. The PSS was originally developed for college student samples and prior findings indicate the PSS has acceptable psychometric properties, including good internal consistency (*α*s ranging from .84 to .86) and good two-day but somewhat weaker six-week test-retest reliability (.85 and .55, respectively). In terms of validity, the PSS correlates with a range of behavioral and self-report criteria (life events as predictors of depression, social anxiety, or utilization of health services) in the expected direction [23]. The Hungarian translation also demonstrated acceptable internal consistency (*α*=.88) [25].

In the current sample, internal consistency of the Total PSS was excellent, with a Cronbach’s alpha value of .916. Data were analyzed on the Total PSS.

***Additional anamnesis questions.*** In addition to the PLES and PSS, participants’ mothers were also asked about alcohol and drug use as well as smoking during pregnancy; whether they had a high risk pregnancy; whether their child was born to term (pre-term, to term, or later term); whether there were any complications during pregnancy/ upon giving birth; and whether their child’s weight was average (below average, average, above average). These data were analyzed to characterize the sample.

***Adult Self-Report 18-59)*** [26]***.*** The Adult Self-Report (ASR) is a 126-item self-report questionnaire for adults (ages 18–59) assessing aspects of adaptive functioning and problems. The ASR is comprised of *adaptive functioning scales*: friends, spouse/partner; family; job; education, personal strengths; *DSM-oriented scales*: anxiety, depressive, somatic, avoidant personality, attention-deficit/ hyperactivity (inattention and hyperactivity/impulsivity subscales), and antisocial personality problems; and *syndrome scales*: anxious/depressed, withdrawn, somatic complaints, thought problems, attention problems, aggressive behavior, rule-breaking behavior, and intrusive behavior; as well as items assessing substance use (alcohol, drugs, and tobacco). Adaptive functioning items and problem items give a Total Problems score as well as the Externalizing and Internalizing Problems broadband scale scores. Respondents rate items are rated on a 3-point scale (0 – ‘Not True’, 1 – ‘Somewhat or Sometimes True’, 2 – ‘Very True or often True’).

Prior findings indicate the Adult Self-Report has adequate psychometric properties, including good internal consistency for most scales, with mean alpha coefficients on the ASR of .83 for the empirically based problem scales and .78 for the DSM-oriented scales. Evidence also indicates the Adult Self-Report exhibited good 1-week test-retest reliability (*r*s as large as .80 for most scales, with none<.71) and content validity (as indexed, e.g., by the problem items discriminating between non-referred and referred samples) [27].

For purposes of the current study, the English version of the Adult Self-Report was translated into Hungarian following the same procedures as for the RST-PQ. The publisher approved the translated measure (Achenbach System of Empirically Based Assessment (ASEBA) representative, personal communication, August 26, 2019). In the current study, data were analyzed on the Internalizing Problems subscale.

**Results**

**Mediation Analyses with the PLES as the Predictor**

***Negative Affectivity as the Outcome***

CATS neglect/negative atmosphere and BIS sensitivity mediated the association between PLES and NA (effect=.062; SE=.020; 95%CIs [.025;.105]). Greater PLES was associated with greater CATS neglect/negative atmosphere and higher scores on CATS neglect/negative atmosphere were associated with greater BIS sensitivity which, in turn, was positively associated with NA. CATS neglect/negative atmosphere was also positively associated with NA but the association between PLES and NA was not significant. Jointly, PLES, CATS neglect/negative atmosphere, and BIS sensitivity accounted for 51% of the variance in NA (Table S4). Indirect effect 1 was (effect=.044; SE=.017; 95%CIs [.014;.082]), but Indirect effect 2 was not (effect=.006; SE=.030; 95%CIs [-.052;.066]) supported.

***Emotion Dysregulation as the Outcome***

CATS neglect/negative atmosphere and BIS sensitivity mediated the association between PLES and DERS (effect=.063; SE=.020; 95%CIs [.026;.105]). Greater PLES was associated with greater CATS neglect/negative atmosphere and higher scores on CATS neglect/negative atmosphere was associated with greater BIS sensitivity which, in turn, was positively associated with greater ED. CATS neglect/negative atmosphere was also associated with greater ED but the associations between PLES and BIS and PLES and ED were not significant. Jointly, PLES, CATS neglect/negative atmosphere, and BIS sensitivity accounted for 52% of the variance in ED (Table S4). Indirect effect 1 was (effect=.040; SE=.017; 95%CIs [.012;.077]), but Indirect effect 2 was not (effect=.006; SE=.031; 95%CIs [-.055;.070]) supported.

***Aggression as the Outcome***

CATS neglect/negative atmosphere and BIS sensitivity mediated the association between PLES and aggression (effect=.024; SE=.013; 95%CIs [.005;.055]). Greater PLES was associated with greater CATS neglect/negative atmosphere and higher scores on CATS neglect/negative atmosphere was associated with greater BIS sensitivity which, in turn, was positively associated with aggression. CATS neglect/negative atmosphere was also positively associated with aggression but the associations between PLES and BIS and PLES and aggression were not significant. Jointly, PLES, CATS neglect/negative atmosphere, and BIS sensitivity accounted for 16% of the variance in aggression (Table S4). Indirect effect 1 was (effect=.048; SE=.023; 95%CIs [.009;.100]), but Indirect effect 2 was not (effect=.003; SE=.013; 95%CIs [-.024;.030]) supported.

**References**

[1] D. Wechsler, “Wechsler intelligence scale for children–Fourth Edition (WISC-IV).” San Antonio, TX: The Psychological Corporation., 2003.

[2] D. Wechsler, “Wechsler adult intelligence scale–Fourth Edition (WAIS–IV).” APA PsycTests., 2008.

[3] B. Sanders and E. Becker-Lausen, “The measurement of psychological maltreatment: Early data on the child abuse and trauma scale,” *Child Abuse and Neglect*, vol. 19, no. 3, pp. 315–323, 1995, doi: 10.1016/S0145-2134(94)00131-6.

[4] A. Láng and K. Lénárd, “The relation between memories of childhood psychological maltreatment and Machiavellianism,” *Personality and Individual Differences*, vol. 77, pp. 81–85, 2015, doi: 10.1016/j.paid.2014.12.054.

[5] P. J. Corr and A. J. Cooper, “The Reinforcement Sensitivity Theory of Personality Questionnaire (RST-PQ): Development and validation,” *Psychological Assessment*, vol. 28, no. 11, pp. 1427–1440, 2016.

[6] G. Pugnaghi, A. Cooper, U. Ettinger, and P. J. Corr, “The psychometric properties of the German language reinforcement sensitivity theory-personality questionnaire (RST-PQ),” *Journal of Individual Differences*, 2018, doi: 10.1027/1614-0001/a000262.

[7] L. J. K. Eriksson, B. Jansson, and Ö. Sundin, “Psychometric properties of a Swedish version of the reinforcement sensitivity theory of personality questionnaire,” *Nordic Psychology*, 2019, doi: 10.1080/19012276.2018.1516563.

[8] D. Watson, L. A. L. A. L. A. Clark, A. Tellegen, A. Tellegan, A. Tellegen, and A. Tellegan, “Development and Validation of Brief Measures of Positive and Negative Affect: The PANAS Scales,” *Journal of Personality and Social Psychology*, vol. 54, pp. 1063–1070, 1988, doi: 10.1037/0022-3514.54.6.1063.

[9] D. Watson, L. A. Clark, and A. Tellegen, “Development and validation of brief measures of positive and negative affect: The PANAS scales.,” *Journal of personality and social psychology*, vol. 54, pp. 1063–1070, 1988, doi: 10.1521/soco_2012_1006.

[10] Á. Gyollai *et al.*, “Psychometric properties of the Hungarian version of the original and the short form of the Positive and Negative Affect Schedule (PANAS).,” *Neuropsychopharmacologia Hungarica : a Magyar Pszichofarmakologiai Egyesulet lapja = official journal of the Hungarian Association of Psychopharmacology*, vol. 13, no. 2, pp. 73–79, 2011.

[11] K. L. Gratz and L. Roemer, “Multidimensional assessment of emotion regulation and dysregulation: Development, factor structure, and initial validation of the Difficulties in Emotion Regulation Scale,” in *Journal of Psychopathology and Behavioral Assessment*, 2004, vol. 26, pp. 41–54. doi: 10.1023/B:JOBA.0000007455.08539.94.

[12] M. Adrian, J. Zeman, C. Erdley, L. Lisa, K. Homan, and L. Sim, “Social contextual links to emotion regulation in an adolescent psychiatric inpatient population: Do gender and symptomatology matter?,” *Journal of Child Psychology and Psychiatry*, vol. 50, no. 11, pp. 1428–1436, 2009, doi: 10.1111/j.1469-7610.2009.02162.x.

[13] C. A. A. Vasilev, S. E. E. Crowell, T. P. P. Beauchaine, H. K. K. Mead, and L. M. M. Gatzke-Kopp, “Correspondence between physiological and self-report measures of emotion dysregulation: A longitudinal investigation of youth with and without psychopathology,” *Journal of Child Psychology and Psychiatry*, vol. 50, no. 11, pp. 1357–1364, 2009, doi: 10.1111/j.1469-7610.2009.02172.x.

[14] A. Weinberg and E. D. D. Klonsky, “Measurement of emotion dysregulation in adolescents,” *Psychological Assessment*, vol. 21, no. 4, pp. 616–621, 2009, doi: 10.1037/a0016669.

[15] N. Bunford, S. W. Evans, and J. M. Langberg, “Emotion Dysregulation Is Associated With Social Impairment Among Young Adolescents With ADHD,” *Journal of Attention Disorders*, vol. 22, no. 1, pp. 66–82, 2018, doi: 10.1177/1087054714527793.

[16] N. Bunford, S. W. Evans, S. P. Becker, and J. M. Langberg, “Attention-Deficit/Hyperactivity Disorder and Social Skills in Youth: A Moderated Mediation Model of Emotion Dysregulation and Depression,” *Journal of Abnormal Child Psychology*, vol. 43, no. 2, pp. 283–296, 2015, doi: 10.1007/s10802-014-9909-2.

[17] G. Kökönyei, R. Urbán, M. Reinhardt, A. Józan, and Z. Demetrovics, “The difficulties in emotion regulation scale: Factor structure in chronic pain patients,” *Journal of Clinical Psychology*, vol. 70, no. 6, pp. 589–600, 2014, doi: 10.1002/jclp.22036.

[18] A. H. Buss and M. Perry, “The aggression questionnaire,” *Journal of Personality and Social Psychology*, vol. 63, no. 3, pp. 452–459, 1992.

[19] C. W. Mathias *et al.*, “Characterizing Aggressive Behavior with the Impulsive/ Premeditated Aggression Scale among Adolescents with Conduct Disorder,” *Psychiatry Res*, vol. 151, no. 3, pp. 231–242, 2007.

[20] J. Gerevich, E. Bácskai, and P. Czobor, “The generalizability of the Buss – Perry Aggression Questionnaire,” vol. 16, no. 3, pp. 124–136, 2007, doi: 10.1002/mpr.

[21] G. J. DuPaul, T. J. Power, A. D. Anastopoulos, and R. Reid, *ADHD Rating Scale-5 for Children and Adolescents*. New York - London: The Guilford Press, 2016.

[22] M. Lobel, C. J. C. J. DeVincent, A. Kaminer, and B. A. B. A. Meyer, “The impact of prenatal maternal stress and optimistic disposition on birth outcomes in medically high-risk women,” *Health Psychology*, vol. 19, no. 6, 2000, doi: 10.1037/0278-6133.19.6.544.

[23] S. Cohen, D. A. Tyrrell, and A. P. Smith, “Negative life events, perceived stress, negative affect, and susceptibility to the common cold,” *Journal of Personality and Social Psychology*, vol. 64, no. 1, pp. 131–140, 1993.

[24] S. Cohen, To. Kamarck, and R. Mermelstein, “A Global Measure of Perceived Stress Author ( s ): Sheldon Cohen , Tom Kamarck and Robin Mermelstein Source : Journal of Health and Social Behavior , Vol . 24 , No . 4 ( Dec ., 1983 ), pp . 385-396,” *Journal of Health and Social Behavior*, vol. 24, no. 4, pp. 385–396, 2014.

[25] A. Stauder and B. Konkoly Thege, “Az észlelt stressz kérdőív (PSS) magyar verziójának jellemzői,” *Mentálhigiéné és Pszichoszomatika*, vol. 7, no. 3, pp. 203–216, Sep. 2006, doi: 10.1556/mental.7.2006.3.4.

[26] L. A. Rescorla, T. M. Achenbach, and L. A. Rescorla, “The Achenbach System of Empirically Based Assessment (ASEBA) for Ages 18 to 90 Years.,” 2004.

[27] T. M. Achenbach and L. Rescorla, “Manual for the ASEBA adult forms & profiles: For ages 18-59: Adult self-report and adult behavior checklist,” 2003.

| Supplementary Table S1. | | | |  |
| --- | --- | --- | --- | --- |
| *Number of participants endorsing prenatal events and stressors on the Prenatal Life Events Scale (PLES)* | | | |  |
| Items | *n* (%) responding as “very much” | *n* (%) responding as “moderately” | *n* (%) responding as at least “moderately” | |
| 1. Did you move or look for a new home? | 4 (1.50%) | 8 (3%) | 12 (4.50%) | |
| 2. Did someone important move out of your home? | 2 (0.70%) | 2 (0.70%) | 4 (1.50%) | |
| 3. Did someone move in with you? | 0 | 1 (0.40%) | 1 (0.40%) | |
| 4. Did you live apart from your husband or partner because of job, travel, or other practical reasons? | 9 (3.30%) | 3 (1.10%) | 12 (4.50%) | |
| 5. Did someone important to you other than your husband or partner move away so you didn't see the person as much? | 0 | 1 (0.40%) | 1 (0.40%) | |
| 6. Did you get married or start living with someone as if married? | 0 | 2 (0.70%) | 2 (0.70%) | |
| 7. Did you have extra home or family responsibilities such as caring for an older relative or someone's child? | 4 (1.50%) | 3 (1.10%) | 7 (2.60%) | |
| 8. Did you have unusually big pressures or conflicts at work? | 7 (2.60%) | 15 (5.60%) | 22 (8.20%) | |
| 9. Did you have unusual financial pressures or trouble with money? | 13 (4.80%) | 19 (70%) | 32 (11.80%) | |
| 10. Were you burglarized or robbed? | 3 (1.10%) | 0 | 3 (1.10%) | |
| 11. Did you experience a loss of your house, car, or something else important to you? | 5 (1.90%) | 3 (1.10%) | 8 (3%) | |
| 12. Did anyone close and important to you die? | 11 (4.10%) | 1 (0.40%) | 12 (4.50%) | |
| 13. Did you have serious arguments several times with someone? | 32 (11.90%) | 12 (4.40%) | 44 (16.30%) | |
| 14. Were you in a hurricane, fire or other major disaster? | 0 | 0 | 0 | |
| 15. Did you experience discrimination or harassment because of your race or because you are a woman? | 2 (0.70%) | 0 | 2 (0.70%) | |
| 16. Were you involved in a serious motor vehicle accident? | 0 | 0 | 0 | |
| 17. Did you have serious problems in your sexual relationship? | 3 (1.10%) | 2 (.70%) | 5 (1.80%) | |
| 18. Were you threatened with physical harm by anyone? | 1 (0.40%) | 0 | 1 (0.40%) | |
| 19. Got fired or laid off from work? | 6 (2.20%) | 0 | 6 (2.20%) | |
| 20. Looked for work for 3 weeks or more? | 1 (0.40%) | 1 (0.40%) | 2 (0.80%) | |
| 21. Had trouble with the Department of Social Services? | 0 | 0 | 0 | |
| 22. Was arrested by the police, had problems with the law or immigration, or was in jail? | 0 | 0 | 0 | |
| 23. Was mugged or personally assaulted? | 2 (0.70%) | 1 (0.40%) | 3 (1.10%) | |
| 24. Has a serious physical injury, illness, or hospitalization? | 18 (6.70%) | 9 (3.30%) | 27 (10%) | |
| 25. Had a problem with alcohol or drugs? | 1 (0.40%) | 1 (0.40%) | 2 (0.70%) | |
| 26. Had a serious nervous or emotional problem besides drinking or drugs? | 2 (0.70%) | 0 | 2 (0.70%) | |
| 27. Separated from a spouse or partner because of not getting along? | 10 (3.70%) | 0 | 10 (3.70%) | |
| 28. Got divorced? | 6 (2.20%) | 0 | 6 (2.20%) | |
| 29. Did any other serious events happen during your pregnancy? | Yes: 63 (23.51%) | | | |

| Supplementary Table S2. | | | | |
| --- | --- | --- | --- | --- |
| *Number of participants endorsing prenatal events and stressors on the Perceived Stress Scale (PSS)* | | | | |
| Items | *n* (%) responding as “very often” | *n* (%) responding as “fairly often” | *n* (%) responding as at least “fairly often” |  |
| 1. been upset because of something that happened unexpectedly? | 8 (30%) | 39 (14.40%) | 47 (17.40%) |  |
| 2. felt that you were unable to control the important things in your life? | 6 (2.20%) | 24 (8.90%) | 30 (11.10%) |  |
| 3. felt nervous and “stressed”? | 5 (1.90%) | 36 (13.30%) | 41 (15.20%) |  |
| 4. Dealt successfully with irritating life hassles?* | 6 (2.20%) | 5 (1.90%) | 11 (4.20%) |  |
| 5. felt that you were effectively coping with important changes that were occurring in your life?* | 2 (0.70%) | 9 (3.30%) | 11 (4.20%) |  |
| 6. felt confident about your ability to handle your personal problems?* | 2 (0.70%) | 7 (2.60%) | 9 (3.40%) |  |
| 7. felt that things were going your way?* | 2 (0.70%) | 11 (4.10%) | 13 (4.90%) |  |
| 8. found that you could not cope with all the things that you had to do? | 5 (1.90%) | 35 (130%) | 40 (14.90%) |  |
| 9. been able to control irritations in your life?* | 1 (0.40%) | 3 (1.10%) | 4 (1.50%) |  |
| 10. felt that you were on top of things? * | 3 (1.10%) | 18 (6.70%) | 21 (8%) |  |
| 11. been angered because of things that were outside of your control? | 6 (2.20%) | 34 (12.60%) | 40 (14.80%) |  |
| 12. found yourself thinking about things that you have to accomplish? | 17 (6.30%) | 88 (32.60%) | 105 (38.90%) |  |
| 13. been able to control the way you spend your time?* | 1 (0.40%) | 9 (3.30%) | 10 (3.80%) |  |
| 14. felt difficulties were piling up so high that you could not overcome them? | 6 (2.20%) | 13 (4.80%) | 19 (70%) |  |
| *Note.* * marks reverse-scored items. | | | | |

| Supplementary Table S3. | |
| --- | --- |
| *Number of participants endorsing pre- and perinatal events and stressors on anamnestic questions* | |
| Anamnestic questions | *n*^a^ |
| Inflammatory Disease | 18 (6.72%) |
| Substance Use | 19 (7.09%) |
| High-risk pregnancy | 70 (26.12%) |
| Time of Birth | 70 (26.12%) |
| Weight at Birth | 16 (5.97%) |
| Cry at Birth | 20 (7.46%) |
| Complications During/After Birth | 81 (30.22%) |
| *Note.* Inflammatory disease=maternal sickness involving high fever or inflammation, or infectious disease during pregnancy; Substance use=use of alcohol, cigarettes, or drugs; Time of birth=child was not born to term (i.e., was either pre-term or late term); Weight at birth=child was born with a lower than average birth weight; Cry at birth= child cried immediately at/after birth. ^a^=number of participants who endorsed each item. | |

| Table S4. | | | | | | | | | |
| --- | --- | --- | --- | --- | --- | --- | --- | --- | --- |
| *Model coefficients for serial mediation models testing effects of prenatal maternal stress on the Prenatal Life Events Scale (PLES) through childhood neglect/negative atmosphere and adolescent BIS sensitivity to adolescent affective outcomes* | | | | | | | | | |
|  | Consequent | | | | | | | | |
|  | *M_1_* (CATS NNA) | | | *M_2_* (BIS) | | | *Y* (NA) | | |
| Antecedent | *B* | *b* | SE | *B* | *b* | SE | *B* | *b* | SE |
| *X* (PLES) | .208 | .848*** | .245 | .010 | .064 | .341 | -.019 | -.061 | .131 |
| *M_1_* (CATS NNA) | - | - | - | .505 | .792*** | .092 | .210 | .167*** | .046 |
| *M_2_* (BIS) | - | - | - | - | - | - | .589 | .298*** | .026 |
| Constant | - | 8.947*** | .630 | - | 43.330*** | 1.134 | - | 2.000^§^ | 1.114 |
|  | *R*^2^=.043, *F*(1, 265)=11.953*** | | | *R*^2^=.257, *F*(2, 264)=38.749*** | | | *R*^2^=.513, *F*(3, 263)=93.181*** | | |
|  | Consequent | | | | | | | | |
|  | *M_1_* (CATS NNA) | | | *M_2_* (BIS) | | | *Y* (ED) | | |
| Antecedent | *B* | *b* | SE | *B* | *b* | SE | *B* | *b* | SE |
| *X* (PLES) | .208 | .848*** | .245 | .010 | .064 | .341 | .029 | .301 | .501 |
| *M_1_* (CATS NNA) | - | - | - | .505 | .792*** | .092 | .195 | .493** | .148 |
| *M_2_* (BIS) | - | - | - | - | - | - | .600 | .967*** | .082 |
| Constant | - | 8.947*** | .630 | - | 43.330*** | 1.34 | - | 23.808*** | 3.438 |
|  | *R*^2^=.043, *F*(1, 265)=11.953*** | | | *R*^2^=.257, *F*(2, 264)=38.749*** | | | *R*^2^=.523, *F*(3, 263)=98.032*** | | |
|  | Consequent | | | | | | | | |
|  | *M_1_* (CATS NNA) | | | *M_2_* (BIS) | | | *Y* (aggression) | | |
| Antecedent | *B* | *b* | SE | *B* | *b* | SE | *B* | *b* | SE |
| *X* (PLES) | .203 | .819** | .246 | .012 | .077 | .343 | -.032 | -.253 | .484 |
| *M_1_* (CATS NNA) | - | - | - | .508 | .803*** | .094 | .237 | .467* | .198 |
| *M_2_* (BIS) | - | - | - | - | - | - | .234 | .292** | .107 |
| Constant | - | 8.930*** | .634 | - | 43.250*** | 1.149 | - | 41.168*** | 4.622 |
|  | *R*^2^=.041, *F*(1, 259)=11.112** | | | *R*^2^=.261, *F*(2, 258)=30.163*** | | | *R*^2^=.164, *F*(3, 257)=12.427*** | | |
| *Note. ****: *p*<.001; **: *p*<.01; *: *p*<.05; ^§^: .1>*p*<.05; *B*=standardized regression coefficients*; b*=unstandardized coefficients; SE=heteroscedasticity-consistent standard error estimator. | | | | | | | | | |

1. The RSTP-Q is the revised and updated version of the Carver and White BIS/BAS scales. [↑](#footnote-ref-1)
